# Recent Duplication and Functional Divergence in Parasitic Nematode Levamisole-Sensitive Acetylcholine Receptors

**DOI:** 10.1371/journal.pntd.0004826

**Published:** 2016-07-14

**Authors:** Thomas B. Duguet, Claude L. Charvet, Sean G. Forrester, Claudia M. Wever, Joseph A. Dent, Cedric Neveu, Robin N. Beech

**Affiliations:** 1 Institute of Parasitology, McGill University, Sainte-Anne-de-Bellevue, Quebec, Canada; 2 INRA, UMR1282 Infectiologie Animale et Santé Publique, Nouzilly, France; 3 Université François Rabelais de Tours, UMR1282, Infectiologie Santé Publique, Tours, France; 4 Faculty of Science, University of Ontario Institute of Technology, Oshawa, Ontario, Canada; 5 Department of Biology, McGill University, Montreal, Quebec, Canada; 6 Centre for Host-Parasite Interactions, Ste-Anne-de-Bellevue, Quebec, Canada; University of Melbourne, AUSTRALIA

## Abstract

Helminth parasites rely on fast-synaptic transmission in their neuromusculature to experience the outside world and respond to it. Acetylcholine plays a pivotal role in this and its receptors are targeted by a wide variety of both natural and synthetic compounds used in human health and for the control of parasitic disease. The model, *Caenorhabditis elegans* is characterized by a large number of acetylcholine receptor subunit genes, a feature shared across the nematodes. This dynamic family is characterized by both gene duplication and loss between species. The pentameric levamisole-sensitive acetylcholine receptor has been characterized from *C*. *elegans*, comprised of five different subunits. More recently, cognate receptors have been reconstituted from multiple parasitic nematodes that are found to vary in subunit composition. In order to understand the implications of receptor composition change and the origins of potentially novel drug targets, we investigated a specific example of subunit duplication based on analysis of genome data for 25 species from the 50 helminth genome initiative. We found multiple independent duplications of the *unc-29*, acetylcholine receptor subunit, where codon substitution rate analysis identified positive, directional selection acting on amino acid positions associated with subunit assembly. Characterization of four gene copies from a model parasitic nematode, *Haemonchus contortus*, demonstrated that each copy has acquired unique functional characteristics based on phenotype rescue of transgenic *C*. *elegans* and electrophysiology of receptors reconstituted in *Xenopus* oocytes. We found evidence that a specific incompatibility has evolved for two subunits co-expressed in muscle. We demonstrated that functional divergence of acetylcholine receptors, driven by directional selection, can occur more rapidly than previously thought and may be mediated by alteration of receptor assembly. This phenomenon is common among the clade V parasitic nematodes and this work provides a foundation for understanding the broader context of changing anthelmintic drug targets across the parasitic nematodes.

## Author Summary

Parasitic nematodes pose a global threat to human health and severely impact livestock animals and crops. Most anthelmintic drugs paralyze worms, targeting pentameric neurotransmitter receptors in the neuromuscular synapse. A detailed understanding of this signaling allows the most effective use of existing drugs and the best opportunities for developing new treatments. The model nematode, *Caenorhabditis elegans* has allowed major advances in our understanding of neurotransmitter receptors, including acetylcholine receptors targeted by the drug levamisole. Characterization of equivalent receptors in parasitic nematodes has revealed changes in receptor composition, the consequences of which are not yet clear. The aim of this study was to examine the mechanisms that produce new receptor subunits and characterize changes in receptor function that arise. We identified multiple duplications of *unc-29*, a subunit of the levamisole-sensitive acetylcholine receptor in parasitic nematodes closely related to *C*. *elegans*. We show the parasite, *Haemonchus contortus*, meets the criteria for a good model of this process and demonstrate that new copies of *unc-29* experience selective pressure to rapidly acquire new functional properties, leading to the possibility of new drug targets. An evolved change in compatibility between receptor subunits appears to play a major role in determining the changes in receptor composition.

## Introduction

The ability to control movement based on a nervous system is unique to the animal kingdom and is a major target for anthelmintic drugs. The fundamental importance of neuronal signalling is revealed by the fact that members of all branches of the tree of life including archaea, bacteria, fungi, plants and animals produce toxins that specifically inhibit signalling causing pain, paralysis or death. A large pharmaceutical industry has risen around drugs targeting the nervous system to control pain, mood and behaviour in humans as well as to control insect pests that transmit disease and parasitic nematodes that damage crops and cause infection in livestock. A detailed understanding of how the nervous system is controlled is therefore essential for understanding animal behaviour and the search for new drugs to combat disease.

Examination of the evolutionary history of genes associated with neuronal signaling suggests that extensive gene duplication, interspersed with periods of gene loss are a common feature [1]. Understanding the conditions under which gene duplication events persist and the consequences that follow for the resulting gene copies will therefore have major implications for interpretation of these evolutionary patterns. The long evolutionary period since the last major duplication events mean that the physiological state under which these events occurred is no longer available for study. In addition, any sequence change leading to functional divergence among copies is obscured by subsequent neutral substitution events. With these limitations, our understanding of the mechanisms involved is limited. Ideally, an examination of genes duplicated recently would provide an opportunity to understand the physiological processes involved and limit sequence divergence allowing the cause of functional changes to be identified.

Control of body muscle contraction in the nematode *Caenorhabditis elegans* has been examined in detail. The pentameric ligand-gated ion-channel family of neurotransmitter receptors mediate fast, synaptic signaling in *C*. *elegans* and in the great majority of animals. Five related, or identical, subunits combine into a pentamer with a large extracellular domain where the activating neurotransmitter binds at the interface between two subunits. The receptor is embedded in the post-synaptic membrane by four transmembrane (TM) regions in each subunit. The second TM regions combine to produce a barrier, gating the flow of either anions or cations. Three classes of pLGIC play a predominant role in the neuromuscular synapse of *C*. *elegans*: two stimulatory acetylcholine (ACh) receptors, one sensitive to nicotine (N-AChR), the other sensitive to levamisole (L-AChR), and one inhibitory GABA receptor (UNC-49) [2]. The functional balance between muscle contraction and relaxation in nematodes, leads to sinusoidal movement and results in locomotion. The N-AChR is a homopentamer of the ACR-16 subunit in *C*. *elegans* [3] and orthologs can be found in a variety of other parasitic nematode species representing different clades. This strong conservation is in contrast to the L-AChR that is an obligate heteropentamer in *C*. *elegans*, requiring five different genes contributing subunits [4].

The nematodes, along with insects, are members of an ancient group of organisms, the ecdysozoa, that are characterized by larval stages proceeding through a series of moults [5]. Analysis of protein sequence evolution has been used to divide the nematodes into five primary clades, with the clade I nematodes basal within the phylogeny, and the other clades diverging progressively with clade V nematodes appearing most recently [6–8]. Diversification of the nematodes is characterized by multiple, independent examples of significant lifestyle change, and adaptation to parasitism that might be expected to coincide with a modification of neuromuscular control [9].

Diversification of L-AChR subunits predates the common, ecdysozoan, ancestor, but duplication, divergence and loss of subunits has continued throughout the evolution of nematodes [10, 11]. Reconstitution of the L-AChR from *C*. *elegans* led to a renewed interest in cognate receptors from the parasitic nematodes [12]. Functional AChRs have been reported for the pig parasites *Ascaris suum*, *Oesophagostomum dentatum* and the sheep parasite, *Haemonchus contortus*. Whereas the L-AChR of *C*. *elegans* has a fixed composition, the parasitic nematodes have lost specific subunits and functional receptors, with different pharmacology, have been produced with different subunit combinations with each species [13–15]. Of specific interest, orthologs of the essential *Cel-unc-29* L-AChR subunit gene are present as four copies in the trichostrongylid nematodes, *Haemonchus contortus*, *Teladorsagia circumcincta* and *Trichostrongylus colubriformis* [16]. Importantly, these *unc-29* paralogs have appeared since divergence from the Rhabditid nematodes within clade V. Sequence divergence among the *unc-29* paralogs reported in these parasitic species varies from 20–25% and is similar to divergence from *C*. *elegans*. This recent duplication event therefore represents an attractive system in which to investigate the basis for such gene duplication and functional divergence.

We propose recent diversification of *unc-29* subunits within the clade V nematodes as a model system that will reveal the physiological conditions that increase the likelihood of gene duplication and functional divergence and provide insight into the mechanisms by which such functional adaptation occurs. We show that this system meets three necessary criteria for an appropriate model, that duplication of *unc-29* is a common feature within the clade V nematodes, that the different copies of *unc-29* have diverged in function and that their sequences are sufficiently similar for individual sites under selection during functional divergence to be identified.

Using this system we have shown that duplication of *unc-29* is a common, recurrent feature of the clade V parasitic nematodes and must therefore be taken into account to understand the action of levamisole (LEV) more generally. Functional divergence to produce receptors with novel pharmacology is rapid and driven by directional selection. A major feature of this process includes changes in subunit compatibility that may help to explain the differences reported for the L-AChRs from different parasitic nematodes.

## Results

### Duplication of *unc-29*

**Fig 1:**
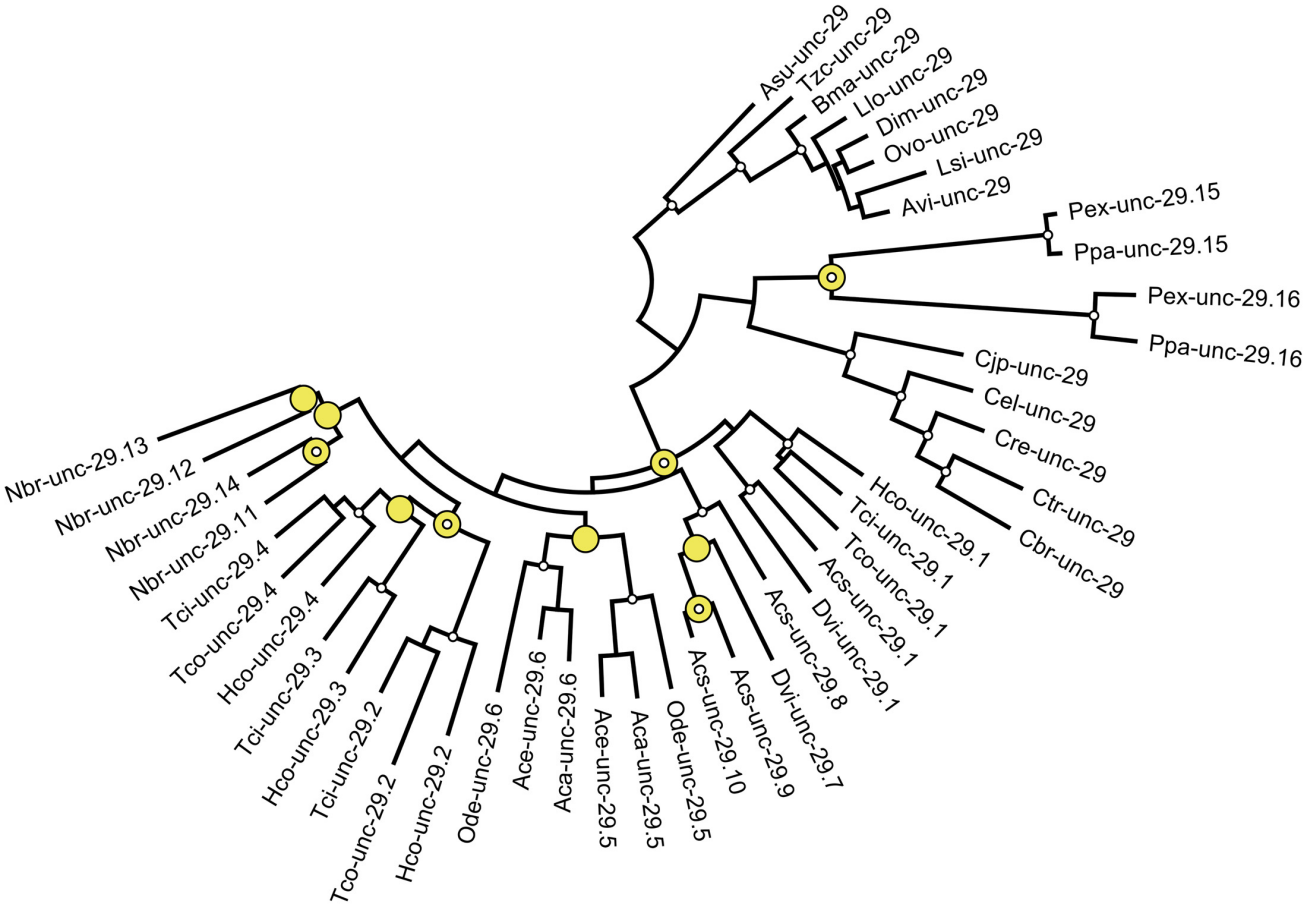
Maximum likelihood phylogeny (PhyML) of *unc-29* codon sequence from clade III and clade V nematodes. Standard nomenclature indicates the species. Asu: *A*. *suum*, Tzc: *T*. *callipaeda*, Bma: *B*. *malayi*, Llo: *L*. *loa*, Dim: *D*. *immitis*, Ovo: *O*. *volvulus*, Lsi: *L*. *sigmodontis*, Avi: *A*. *viteae*, Cjp: *C*. *japonica*, Cel: *C*. *elegans*, Cre, *C*. *remanei*, Ctr, *C*. *tropicalis*, Cbr, *C*. *briggsae*, Pex: *P*. *exspectatus*, Ppa: *P*. *pacificus*, Hco: *H*. *contortus*, Tci: *T*. *circumcincta*, Tco: *T*. *colubriformis*, Acs: *A*. *costaricensis*, Dvi: *D*. *viviparus*, Ode: *O*. *dentatum*, Aca: *A*. *caninum*, Ace: *A*. *ceylenicum*, Nbr: *N*. *brasiliensis*. Reliable nodes with SH support of more than 0.9 are indicated by a white circle. Nodes corresponding to inferred duplication events are indicated by a yellow circle. doi:10.1016/j.pt.2010.04.003, 10.1093/sysbio/syq010

**Fig 2:**
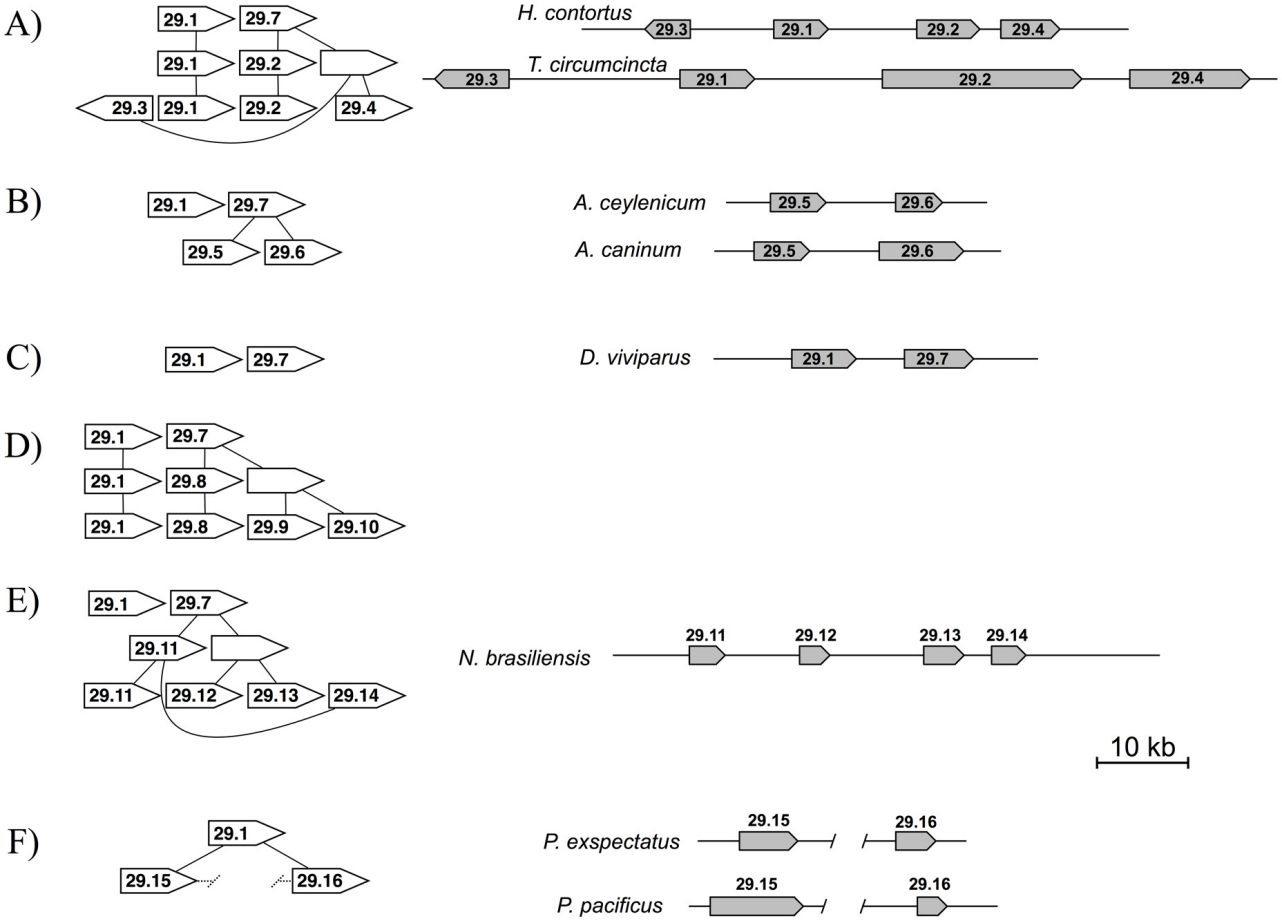
Physical map and conceptual origin for different *unc-29* duplication events. Panels A) to F) correspond to different paralogous families. An idealized history for each family is indicated to the left. A physical map for different species is shown to the right, based on genome data from WormBase ParaSite (WBPS2) or the Sanger Center haemonchus_V1 genome build from 2012. Arrows indicate extent of the gene from start to stop codon, where differences in length occur in introns. Direction of transcription is indicated for each gene and maps are drawn to scale, as indicated.

Cloned cDNA sequences available in Genbank [17] and genomes available in the Wormbase ParaSite WBPS2 database [18] were used to identify homologs of the *C*. *elegans unc-29* gene in different nematode species representing clades III and V [6, 8]. Apparently unique copies of *unc-29* with almost complete coding sequence were identified in each clade III nematode species, *Ascaris suum*, *Thelazia callipaeda*, *Brugia malayi*, *Loa loa*, *Dirofilaria immitis*, *Onchocerca volvulus*, *Litomosoides sigmodontis* and *Acanthocheilonema viteae* and each clade V Rhabditoidea, *Caenorhabditis elegans*, *C*. *japonica*, *C*. *briggsae*, *C*. *remanei* and *C*. *tropicalis*, examined. In contrast, multiple copies of *unc-29* were identified in every member of the *Strongylida* and *Diplogasterida*, including, *Haemonchus contortus*, *Teladorsagia colubriformis*, *Trichostrongylus colubriformis*, *Dictyocaulus viviparus*, *Angiostrongylus costaricensis*, *Ancylostoma caninum*, *Ancylostoma ceylanicum*, *Nippostrongylus brasiliensis*, *Pristionchus pacificus* and *Pristionchus exspectatus*. Fig 1 presents a maximum likelihood phylogeny of these *unc-29* sequences. The genomic organization and inferred events leading to the duplicate genes are shown in Fig 2. Combining the reconstructed phylogeny with their genetic maps suggests at least ten duplication events, one within the *Diplogasterida* and nine within the *Strongylida* (Fig 1). The nomenclature used in Fig 1 reflects this inferred pattern of duplication [19].

The *unc-29*.*1* clade is basal within the *Strongylida* and the pattern of inferred duplication events implies that *unc-29*.*1* persists in *D*. *viviparus*, *A*. *costaricensis* and the *Trichostrongyloidea*: *H*. *contortus*, *T*. *circumcincta* and *T*. *colubriformis*, but has been lost from the *Strongyloidea O*. *dentatum* and the *Ancylostoma* spp. The original duplicate of *unc-29*.*1* corresponds to *unc-29*.*7* and *D*. *viviparus* retained both *unc-29*.*1* and *unc-29*.*7*. Interestingly, *A*. *costaricensis* contains additional copies derived from *unc-29*.*7*, producing *unc-29*.*8*, *unc-29*.*9* and *unc-29*.*10*. (Fig 2). These also appear to be present in *A*. *cantonensis* but its genome assembly is not sufficiently complete to identify the copies unambiguously. An independent duplication of *unc-29*.*7* produced *unc-29*.*5* and *unc-29*.*6*, shared by *O*. *dentatum*, *A*. *caninum* and *A*. *ceylenicum*. *N*. *brasiliensis* possessed four copies derived from *unc-29*.*7* that were found only within this species. A similar quadruplication of *unc-29*.*7* is inferred to have occurred in the *Trichostrongyloidea* following divergence from *Nippostrongylus* but prior to diversification of the trichostrongylid group to produce *unc-29*.*2*, *unc-29*.*3* and *unc-29*.*4* now shared by *H*. *contortus*, *T*. *circumcincta* and *T*. *colubriformis* [16].

In these representative genomes, a majority of the *unc-29* duplication events were local, within 20kb of the original gene and in the same orientation (Fig 2). Exceptions are *unc-29*.*3* that is found in the opposite orientation to the other three copies in both *H*. *contortus* and *T*. *circumcincta*, and the duplication within the *Diplogasteroidea* to produce *unc-29*.*15* and *unc-29*.*16* that were found dispersed, on different scaffolds, within the genomes of *P*. *pacificus* and *P*. *exspectatus*. It is noteworthy that the phylogeny in Fig 1 and genetic maps in Fig 2 are consistent with each derived copy resulting from an upstream gene.

The many examples of recently duplicated *unc-29* genes and the number of independent duplication events provides an opportunity to examine the nature of selective pressure acting during the duplication and adaptation process.

### Substitution rate analysis

**Fig 3:**
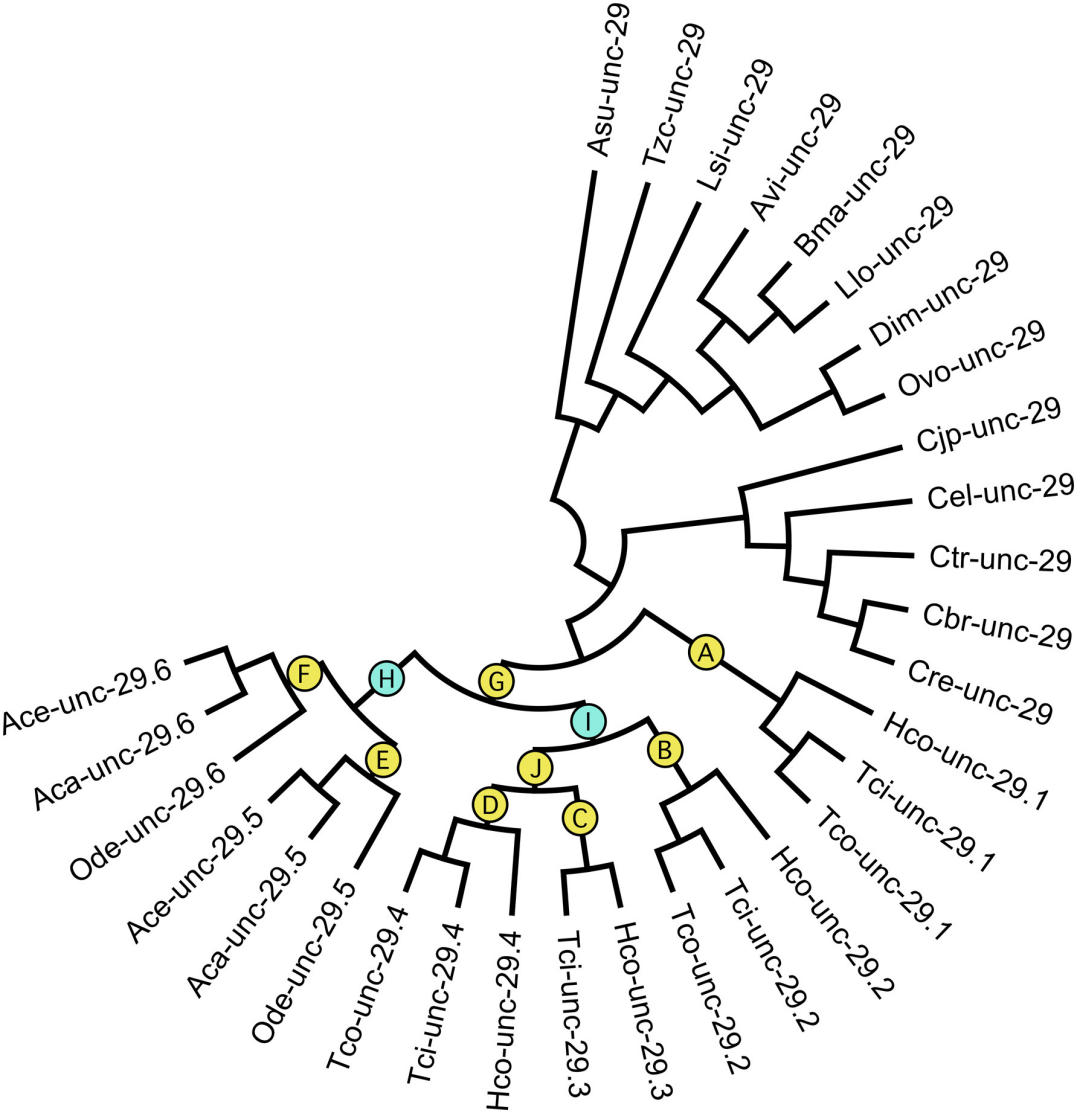
Cladogram of *unc-29* copies. This phylogeny was used to develop codon substitution models that produced the data in Table 1. Branches within the *Strongyloidea*, leading to the different genes are labelled A-J. Branches leading to paralogous copies are labelled yellow, those corresponding to orthologs in different species are labelled blue. See Fig 1 for species names.

The clade III nematodes and the clade V *Rhabditoidea* provide representatives of single copies of *unc-29*. We focused on the paralogous genes *unc-29*.*1* to *unc-29*.*6* because they occur in *H*. *contortus* and *O*. *dentatum*, species where reconstitution of a functional L-AChR has been achieved [14, 15] and where there are multiple representatives of each gene copy available. Our analysis of relative substitution rate was based on the reduced phylogeny shown in Fig 3. Overall protein identity between copies of *unc-29* from Fig 3 range from 92% (*unc-29*.*4* and *unc-29*.*5*) down to 76% (*unc-29*.*2* and *unc-29*.*3*), in comparison to 74% comparing *C*. *elegans unc-29* with *unc-29*.*2*.

Comparison of substitution rates using PAML 4.8 found that branch substitution rates were well below the level at which sites became saturated [20]. A model with three rate classes (M3k3) was a significantly better fit to the data than either two (M3k2) or a single (M0) substitution rate (see Table 1) [21]. A majority (63%) of codons were highly conserved (ω = 0.003), consistent with strong conservation of subunit tertiary structure [22]. A second class of sites (27%) evolved more than ten times faster (ω = 0.042) and a minority (10%) with the fastest rate (ω = 0.212).

**Table 1:** Substitution rate analysis of *unc-29* duplications.

Model	Test	2δ	d.f.	Prob.		Rate Classes			Positively Selected Sites
One Class (M0)					p	1.000			
					ω	0.0315			
Two Class (M3k2)	M3k2—M0	881	2	< 10^−4^	p	0.823	0.177		
					ω	0.0093	0.1510		
Three Class (M3k3)	M3k3—M0	67.2	2	< 10^−4^	p	0.626	0.274	0.099	
					ω	0.0033	0.0430	0.2112	
Branch A (MDa)	MDa—M3k3	42.6	1	< 10^−4^	p	0.599	0.292	0.109	108, 351, 414
					ω	0.0028	0.0383	0.1854	
					ω _branch_			**3.3245**	
Branch B (MDb)	MDb—M3k3	35.7	1	< 10^−4^	p	0.540	0.348	0.112	71, 486
					ω	0.0016	0.0300	0.1992	
					ω _branch_		0.2268		
Branch C (MDc)	MDc—M3k3	17.7	1	< 10^−4^	p	0.631	0.271	0.098	105, 417
					ω	0.0034	0.0435	0.2010	
					ω _branch_			**1.1163**	
Branch D (MDd)	MDd—M3k3	11.8	1	0.0005	p	0.611	0.284	0.105	28, 206, 364, 421
					ω	0.0030	0.0402	0.1986	
					ω _branch_			**1.4148**	
Branch E (MDe)	MDe—M3k3	14.4	1	10^−4^	p	0.620	0.277	0.103	103, 105
					ω	0.0032	0.0418	0.1975	
					ω _branch_			0.9296	
Branch F (MDf)	MDf—M3k3	7.59	1	0.0058	p	0.619	0.280	0.101	103, 105, 364, 400
					ω	0.0032	0.0419	0.2027	
					ω _branch_			**1.4681**	
Branch G (MDg)	MDg—M3k3	28.6	1	< 10^−4^	p	0.645	0.262	0.093	105, 329, 341,
					ω	0.0036	0.0462	0.2047	351, 364, 422, 492
					ω _branch_			**6.1297**	
Branch H (MDh)	MDh—M3k3	1.87	1	0.1714	p	0.621	0.278	0.101	
					ω	0.0032	0.0421	0.2108	
Branch I (MDi)	MDi—M3k3	3.52	1	0.0606	p	0.616	0.281	0.103	
					ω	0.0031	0.0412	0.2031	
Branch J (MDj)	MDj—M3k3	6.07	1	0.0137	p	0.633	0.271	0.097	108, 372, 418, 426
					ω	0.0034	0.0444	0.2111	
					ω _branch_			**4.2753**	

**Fig 4:**
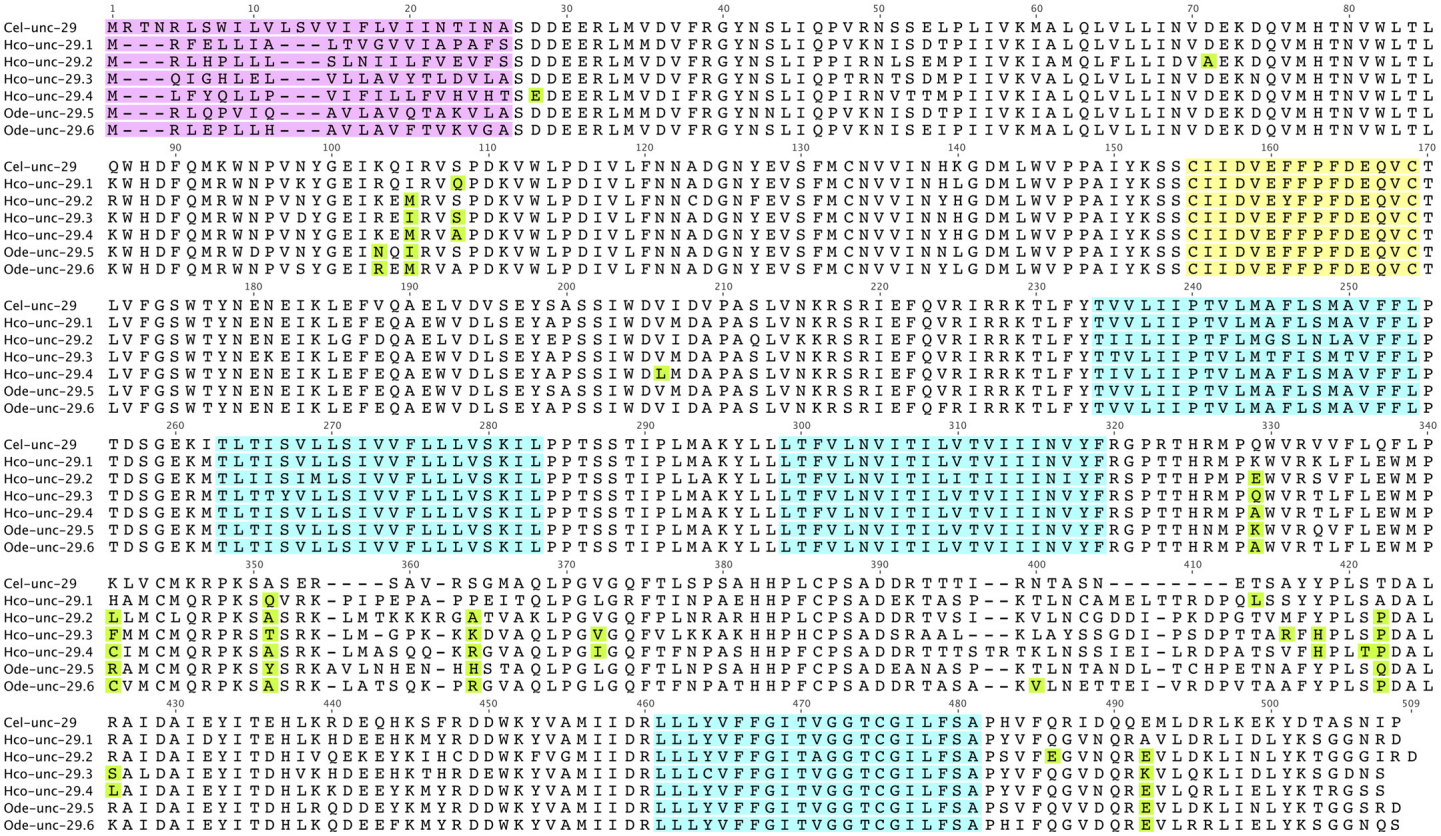
Alignment of UNC-29 protein. Sequence for *C*. *elegans* is shown in reference to six representative paralogs from *H*. *contortus* and *O*. *dentatum*. The signal peptide is indicated in pink, four transmembrane regions in blue and the characteristic cys-loop in yellow. Amino acids under positive selection are indicated in green.

Comparison of different nested substitution rate class models M0 (single rate), M3k2 (two rate), M3k3 (three rate) and MD (three rate branch-site) for each branch labelled (A-J) in Fig 3 and positively selected sites determined by two rate fixed effects likelihood analysis using coordinates given in Fig 4.

The branch-site test, implemented in PAML Model D was applied to ancestral branches within the *Strongyloidea*. Eight of these branches (A-G and J, Fig 3) correspond to divergence following an inferred gene duplication event and two (H and I, Fig 3) to divergence of the *Trichonstrongyloidea* and *Strongyloidea* leading to each copy of *unc-29*. For *unc-29*.*1*, *unc-29*.*3*, *unc-29*.*4* and *unc-29*.*6*, the minority class of fastest evolving sites had significantly higher, branch specific substitution rates above 1, ranging from ω = 1.12 to ω = 3.32, consistent with positive selection, and possibly a shift in subunit function. For *unc-29*.*5*, this rate class also increased significantly (ω = 0.93) consistent with relaxed, neutral evolution. For *unc-29*.*2*, a significant change was observed in the intermediate rate class, rising from ω = 0.030 to 0.227 (Table 1).

It is likely that only a few sites within each rate class were responsible for the overall relative substitution rate increase for each branch. We therefore used a fixed effect likelihood analysis (two rate FEL, HyPhy v 2.2.6) to determine specific amino acid sites under positive selection for branches corresponding to the different duplication events in Fig 3. [23, 24]. Significant positive selection was observed for multiple sites on each branch and a majority of these occurred within the intracellular loop between TM3 and TM4 (Table 1 and Fig 4). This region is known to be involved in interaction with the receptor assembly machinery for AChRs [25], and phosphorylation that regulates assembly [26], receptor localization [27] and channel function [28]. Only one site: 416, coincided with the appearance of a predicted PKC phosphorylation site. Sites 103, 105 and 108 that are selected on multiple branches lie at the interface between subunits on the complementary face approximately 15–20 Å above the ligand binding pocket. They may influence subunit interactions, but none were expected to influence the neurotransmitter binding site. Sites 486 and 492 occur in the C-terminal tail beyond TM 4, a region that has no clearly defined function. Sites 71 and 206 occur within beta-strand regions of the 3D structure and it is not clear how they could affect structure or function of the receptor.

### Hco-UNC-29 copies functionally replace Cel-UNC-29 in transgenic *C*. *elegans*

We chose the four copies of *unc-29* from *H*. *contortus* as representatives of the wider phenomenon of *unc-29* subunit duplication in the clade V nematodes. To evaluate functional divergence of *Hco-unc-29* copies from *C*. *elegans*, we evaluated the ability of each to functionally replace the defective *Cel-unc-29* and rescue sensitivity to LEV in the defining *unc-29* mutant strain, CB1072 [29]. Transgene expression was driven by the *myo-3* promoter that allows expression in body muscles where the native *C*. *elegans* L-AChR is present [2]. Over-expression of transgenes may disturb the normal pattern of intracellular receptor expression and so measurement of normal locomotion was not felt to be an appropriate evaluation of rescue. Instead we focused only on the ability of expressed receptors to conduct ions in the presence of LEV as judged by paralysis on exposure to LEV.

**Fig 5:**
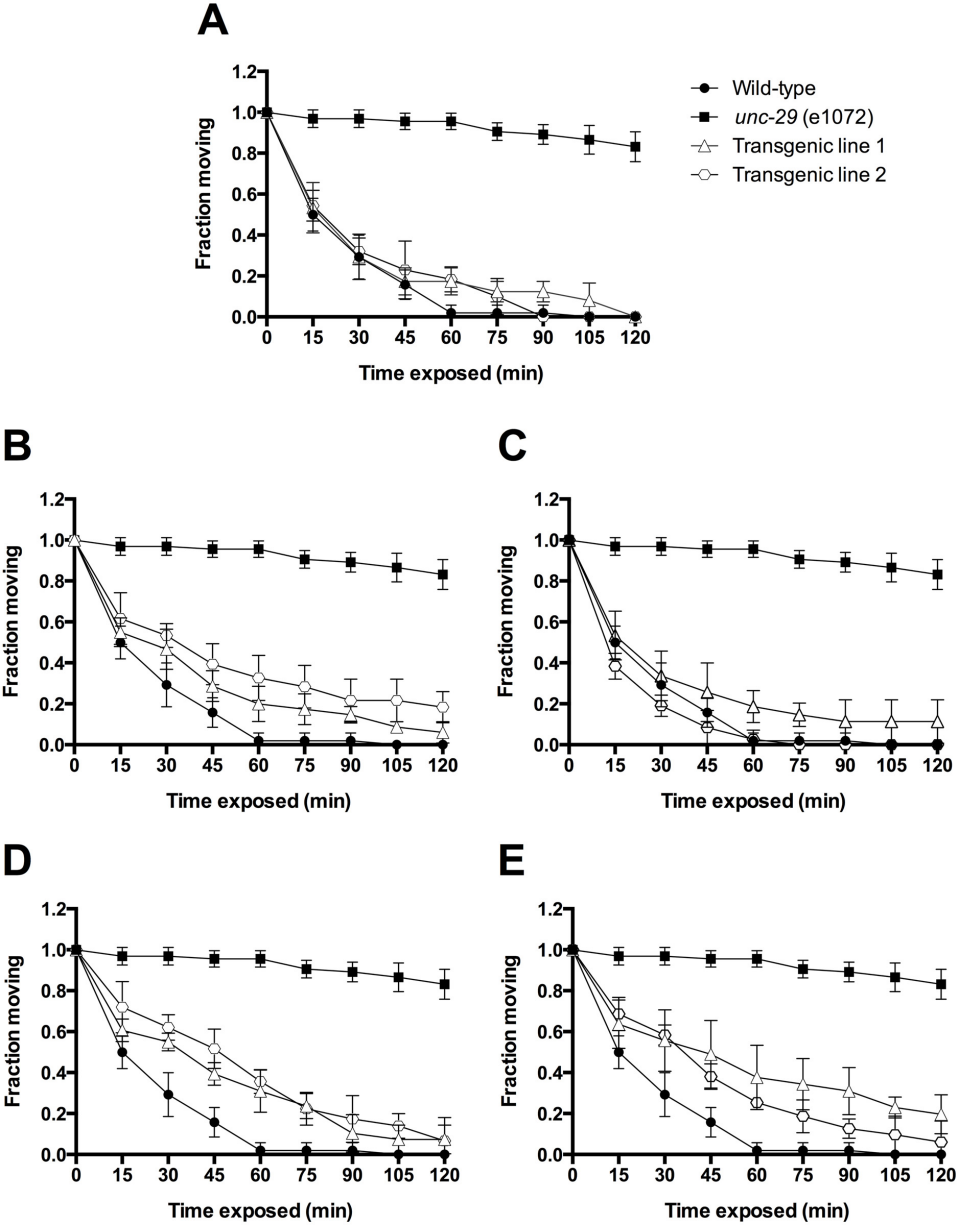
Rescue of LEV sensitivity in transgenic *C*. *elegans*. *Hco-unc-29* gene copies were transfected into *C*. *elegans unc-29 (e1072)* mutant strain (CB1072). The number of moving animals under exposure to 200 μM LEV every 15 min are shown on the Y axes. N2 Bristol wild-type (black circles) and CB1072 *unc-29 (e1072)* (black squares) controls are shown for comparison. Two independent transfected worm lines (white circles and triangles) were examined for each experiment. This assay was repeated three times on 12 animals per worm line. A) Transfection of *Cel-unc-29*. B-E) Transfection of *unc-29*.*1*, *unc-29*.*2*, *unc-29*.*3* and *unc-29*.*4* respectively. Data are plotted as mean ± SD. A Two-way ANOVA with Bonferroni’s multiple comparison post test was performed. In every case, both transfected lines were significantly more LEV sensitive than the CB1072 *unc-29 (e1072)* mutants (p < 0.001).

Wild-type N2 worms showed rapid paralysis that was complete after an hour of exposure to 200 μM LEV, whereas mutant *unc-29(e1072)* animals were barely affected by the drug over two hours of exposure (Fig 5). Transfection with *Cel-unc-29* elicited complete rescue of LEV sensitivity, with paralysis kinetics very similar as wild-type, N2, animals (Fig 5A) [29]. Transfection with each of the four *H*. *contortus unc-29* copies lead to increased LEV sensitivity in the worms that were paralyzed after two hours of exposure to the drug (Fig 5B–5E). These results revealed a conserved ability of the four paralogs to confer LEV sensitivity.

**Fig 6:**
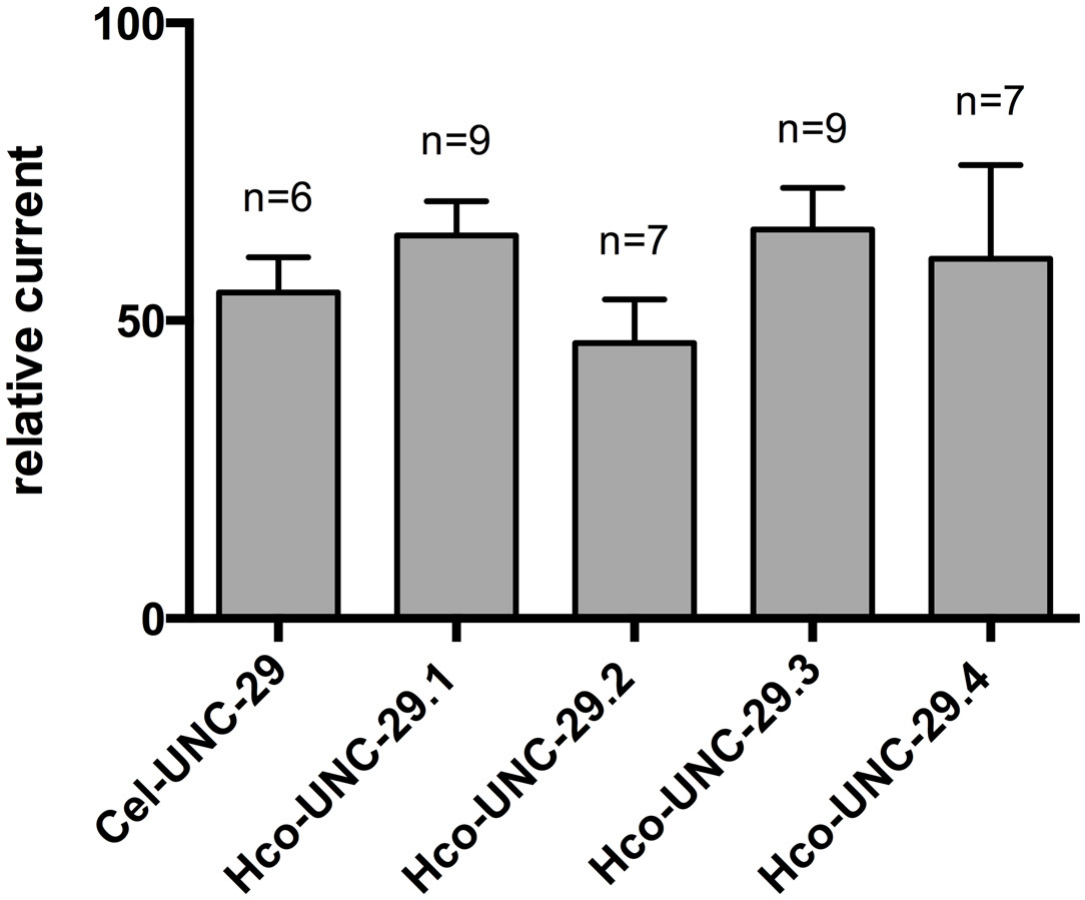
Response of admixture, reconstituted receptors in *Xenopus* oocytes. Two electrode voltage clamp experiments were performed on oocytes injected with *Cel-unc-63*, *Cel-unc-38*, *Cel-lev-8*, *Cel-lev-1* cRNAs and co-expressed with *H*. *contortus* accessory proteins, *ric-3*, *unc-50* and *unc-74*. The addition of cRNAs encoding *Cel-unc-29*, *unc-29*.*1*, *unc-29*.*2*, *unc-29*.*3* or *unc-29*.*4* were tested independently. LEV response values were normalized to those elicited after perfusion of 100 μM ACh. Error bars indicate SD.

To confirm that the LEV sensitivity rescue could be due to the functional incorporation of parasite protein into the *C*. *elegans* L-AChR, we injected into *Xenopus* oocytes, cRNA admixtures of the four essential *C*. *elegans* L-AChR subunits: *Cel-unc-63*, *Cel-unc-38*, *Cel-lev-8* and *Cel-lev-1*, the three *H*. *contortus* accessory proteins: *ric-3*, *unc-50* and *unc-74*, and the *Cel-unc-29* or each *Hco-unc-29* paralog in turn [4, 14]. Reconstitution of the *C*. *elegans* L-AChR with *Cel-unc-29* produced functional channels as previously described, confirming that the *H*. *contortus* accessory proteins are functional between species [15]. Omitting *unc-29* produced no response, so any current produced using parasite genes must involve their incorporation into a functional L-AChR. Perfusion of 100 μM ACh generated currents with each admixture. UNC-29.1 produced an average of 500 μA currents, while the other three produced reproducible currents below 200 nA with UNC-29.2 producing the smallest response on average (S1 Fig). Perfusion of 100 μM LEV also generated currents for each paralog that, relative to the ACh response, were comparable between subunit copies (Fig 6).

All together, these findings confirm that each of the four *H*. *contortus* UNC-29 paralogs can functionally replace Cel-UNC-29. This is consistent with the phenotype rescue observed in transgenic *C*. *elegans* and suggested the rescue was a result of incorporation of the parasite derived *unc-29* into the *C*. *elegans* L-AChR.

### Hco-UNC-29 copies can form new L-AChRs with distinct pharmacological profiles

**Fig 7:**
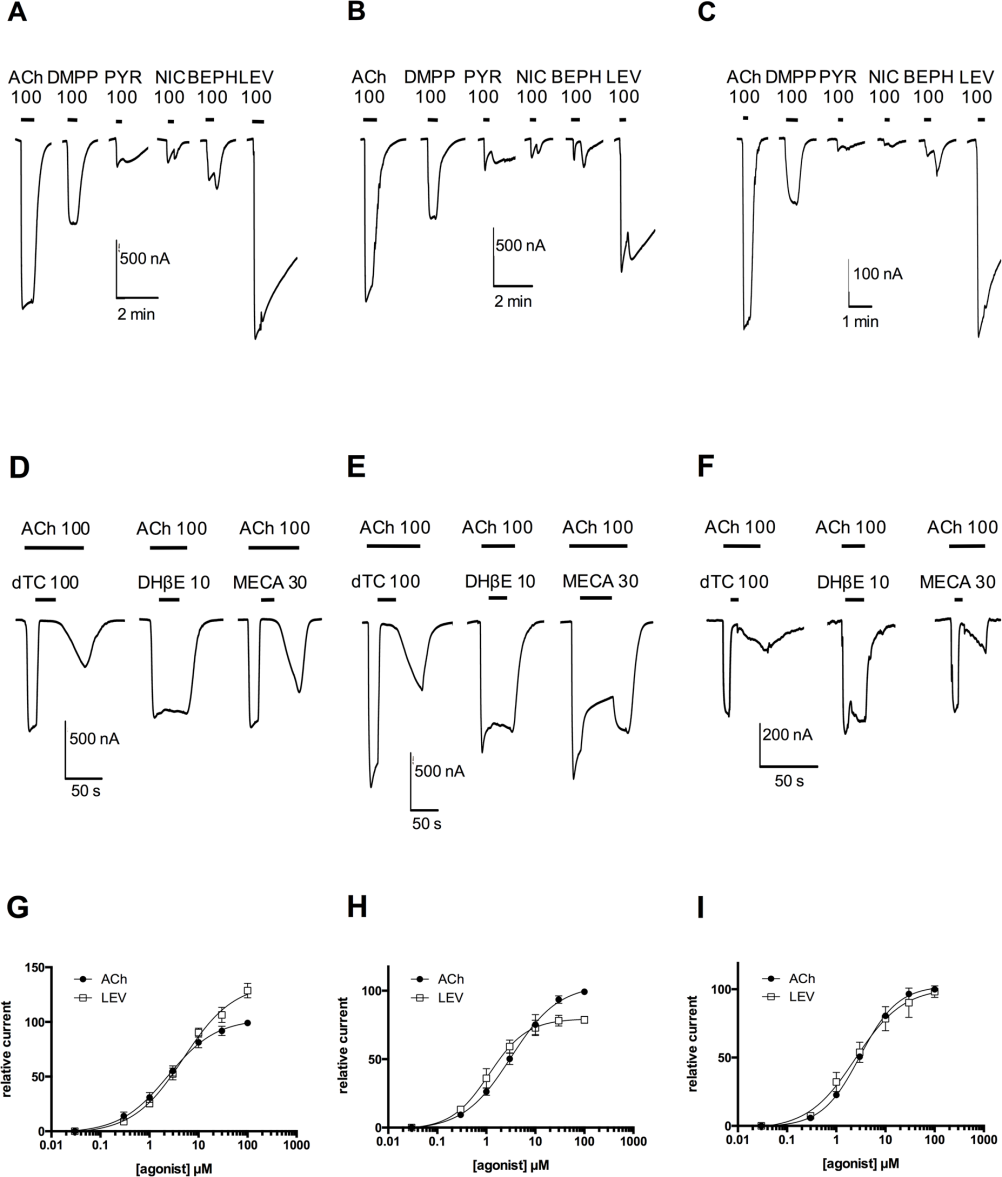
Response of *H*. *contortus* reconstituted receptors in *Xenopus* oocytes. Two electrode voltage clamp experiments were performed on oocytes injected with *Hco-unc-63*, *Hco-unc-38*, *Hco-acr-8*, and *Hco-ric-3*.*1*, *Hco-unc-74*, *Hco-unc-50* cRNAs. *Hco-unc-29*.*1*, *unc-29*.*3* and *unc-29*.*4* were combined independently with the cRNA mixture. A), B) and C) Representative recording traces from single oocytes perfused with 100 μM of the following cholinergic agonists: acetylcholine (ACh), Dimethylpiperazinium (DMPP), Pyrantel (PYR), Nicotine (NIC), Bephenium (BEPH) and Levamisole (LEV). D), E) and F) Representative recording traces from single oocytes continuously perfused with 100 μM ACh. Oocytes were perfused with the following cholinergic antagonists: D-tubocurarine (dTC, 100 μM), Dihydro-β-erythroidine (DHβE, 10 μM) and Mecamylamine (MECA, 30 μM). Black horizontal bars show the time period of agonist and or antagonist application. G), H) and I) Concentration-response curves for the L-AChR1.1, L-AChR1.3 and L-AChR1.4 for ACh (black circles) and LEV (white squares). All responses are normalized to 100 μM ACh, which corresponds to the saturating dose. The ACh and LEV 50% effective concentration (EC_50_) values as well as Hill coefficients are indicated in Table 2. Error bars represent SD.

Duplication of the *unc-29* genes occurred within the context of a functional neurophysiology distinct from that found in the Rhabditidae. Any functional divergence, beginning from UNC-29 proteins with identical sequence, would be determined by their association, and interaction, with their subunit partners. To investigate this possibility we reconstituted the *H*. *contortus* LEV sensitive AChR (L-AChR1) [14] in *Xenopus* oocytes, sequentially replacing UNC-29.1 with each paralog in turn. For convenience we refer to these as L-AChR1.1, L-AChR1.2, L-AChR1.3 and L-AChR1.4, respectively. Reconstitution of the L-AChR1.1 as a reference, produced large inward currents in the μA range for both ACh and LEV at 100 μM, with LEV acting as a superagonist (125.4 ± 13,0% of ACh response) (Fig 7A). The relative affinity, EC_50_, of the receptor to ACh and LEV was 2.4 ± 0.2 μM and 4.9 ± 0.4 μM respectively (Fig 7G, Table 2), consistent with results described previously [14].

**Table 2:** ACh and LEV response profiles of the reconstituted L-AChR1.1, L-AChR1.3 and L-AChR1.4.

	ACh			LEV		
	EC_50_ (μM)	Hill	n	EC_50_ (μM)	Hill	n
L-AChR1.1	2.38 ± 0.16	0.88 ± 0.05	8	4.91 ± 0.44	0.84 ± 0.05	8
L-AChR1.3	3.19 ± 0.22	0.89 ± 0.05	7	1.15 ± 0.08	1.13 ± 0.09	6
L-AChR1.4	3.03 ± 0.04	1.12 ± 0.04	4	2.48 ± 0.35	0.90 ± 0.12	4

Surprisingly, replacing UNC-29.1 by UNC-29.2 failed to produce any detectable current in response to either 100 μM perfusion of ACh or LEV (S2 Fig). This was repeated with at least three independent batches of oocytes without exhibiting any signal (n = 18), despite the same *unc-29*.*2* cRNA leading to measurable current in combination with *C*. *elegans* subunits.

In contrast, substitution of UNC-29.1 by either UNC-29.3 or UNC-29.4 generated either large (Fig 7B), or smaller (Fig 7C) inward currents, respectively, in response to 100 μM ACh. This confirmed that both UNC-29.3 and UNC-29.4 can participate in the formation of new, functional, L-AChRs. For L-AChR1.3, perfusion of 100 μM LEV produced smaller currents than ACh, with an average of 65.2 ± 9.9% of the ACh current making LEV a partial agonist (Fig 7B). L-AChR1.4 produced similar currents for both ACh and LEV (Fig 7C). Dose-response curves for all three receptors were similar for ACh, with an EC_50_ of approximately 3.2 μM (Fig 7G, 7H and 7I, Table 2). Affinity of L-AChR1.3 for LEV was significantly higher with an EC_50_ of 1.15 ± 0.1 μM compared to the other receptors (Fig 7H, Table 2).

The pharmacological profile of the L-AChR1.1, L-AChR1.3 and L-AChR1.4 was also expanded to other cholinergic agonists (DMPP, nicotine) and anthelmintics (pyrantel, bephenium). In each case, the rank order for these drugs, normalized to the response to 100 μM ACh, was LEV > DMPP >>> Bephenium ~ Pirantel ~ Nicotine. (Fig 7D, 7E and 7F) (Table 3, S3 Fig).

**Table 3:** Pharmacological response of L-AChR1.1, L-AChR1.3 and L-AChR1.4.

	L-AChR1.1		L-AChR1.3		L-AChR1.4	
**Agonists**	**Max. Current ± S.E.**	**n**	**Max. Current ± S.E.**	**n**	**Max. Current ± S.E.**	**n**
Acetylcholine	100	12	100	11	100	8
DMPP	41.98 ± 1.41	9	36.93 ± 3.93	8	31.63 ± 1.00	8
Pyrantel	11.85 ± 0.99	12	13.79 ± 1.26	9	7.32 ± 0.98	7
Nicotine	4.70 ± 1.05	12	6.79 ± 1.46	8	1.52 ± 0.47	6
Bephenium	13.92 ± 1.70	12	9.59 ± 0.83	7	6.45 ± 0.84	7
Levamisole	125.4 ± 3.76	12	65.18 ± 3.50	8	94.71 ± 2.93	8
**Antagonists**	**Max. Inhibition ± S.E.**	**n**	**Max Inhibition ± S.E.**	**n**	**Max Inhibition ± S.E.**	**n**
D-turbocurarine	99.63 ± 0.35	11	99.98 ± 0.02	6	97.52 ± 1.09	7
DHßE	15.98 ± 2.16	11	23.55 ± 1.94	7	16.51 ± 5.73	6
Mecamylamine	99.91 ± 0.09	8	56.87 ± 3.18	5	96.4 ± 1.92	8

The response to a variety of agonists and antagonists was normalized to those elicited by 100 μM acetylcholine with n indicating the number of oocytes evaluated.

We also tested activity of the cholinergic antagonists d-tubocurarine (dTC), dihydro-β-erythroidine (DHβE) and mecamylamine (MECA) on the reconstituted L-AChR1.1, L-AChR1.3 and L-AChR1.4. The application of 100 μM of the canonical antagonist dTC efficiently blocked the ACh responses (Fig 7D, 7E and 7F, Table 3). Moreover, there was also evidence that all three receptors responded similarly to the competitive nicotinic antagonist DHβE, showing weak average inhibition. However, L-AChR1.3, surprisingly, responded very differently to the allosteric antagonist MECA, which exhibited complete blocking of ACh elicited currents for L-AChR1.1 (99.9 ± 0.1%) and L-AChR1.4 (96.4 ± 1.9%) (Fig 7D and 7F, Table 3) but only partially antagonized ACh-evoked currents of L-AChR1.3 (56.9 ± 3.2%) (Fig 7E, Table 3).

In summary, two novel receptors, L-AChR1.3 and L-AChR1.4, with high affinity to ACh and LEV, were reconstituted in *Xenopus* oocytes in addition to the previously identified L-AChR1.1 Overall, each of the four paralogs of *unc-29* displayed a unique functional characteristic, either in their (in)ability to reconstitute an ACh responsive channel, or in the agonist characteristics of LEV and response to the antagonist Meca.

### Hco-UNC-29.1 and Hco-UNC-29.2 are co-expressed in muscle tissue

There are many possible fates for duplicate copies of a gene that include changes in functional characteristics as well as altered patterns of expression [30]. Given that both UNC-29.1 and UNC-29.2 were capable of assembling into a functional L-AChR *in vitro* and *in vivo* with *C*. *elegans* subunits, they may have the potential to co-assemble, or interfere with each other, if co-expressed in the same tissue *in vivo* in *H*. *contortus*. We raised antibodies against peptides, specific for either UNC-29.1 or UNC-29.2, in the highly variable intracellular loop between TM3 and TM4. Specificity and cross-reactivity of the antibodies was verified by an ELISA assay for binding to the antigen peptides and to native protein expressed in *Xenopus* oocytes. Antibodies against both UNC-29.1 and UNC-29.2 were specific and showed fluorescence at the oocyte surface only with their cognate receptor (S4 Fig). Positive fluorescence was blocked by pre-absorption with specific peptide antigen.

**Fig 8:**
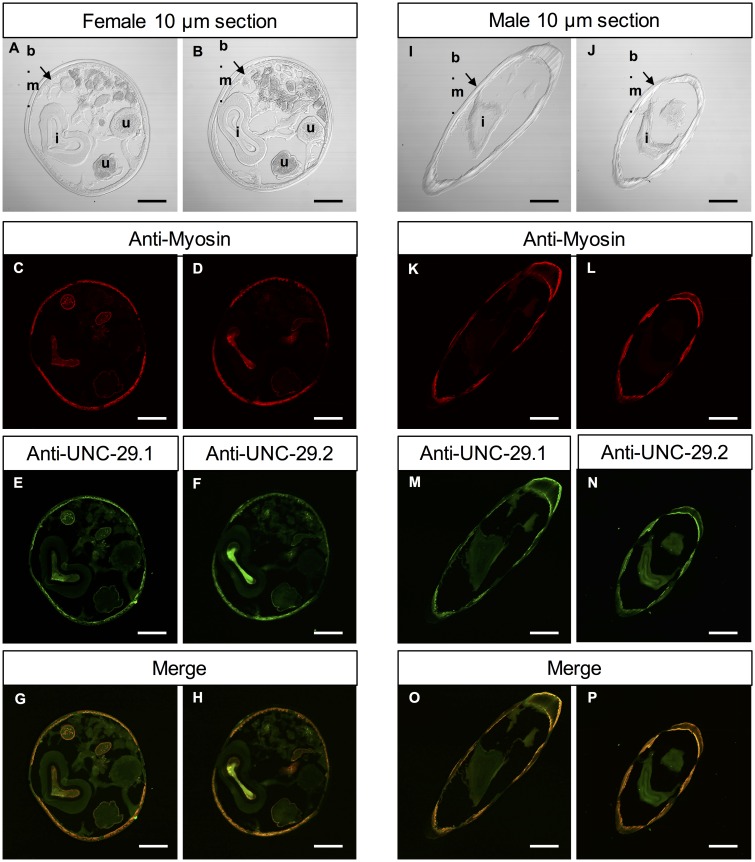
Immunolocalization of UNC-29.1 and UNC-29.2 in adult *H*. *contortus*. Adults were fixed and incubated with affinity-purified antibodies raised against UNC-29.1 or UNC-29.2 specific peptides. The localization of both subunits were performed using Alexa 488-labeled secondary antibodies (green). A–H) Confocal microscopy performed on female 10 μm transversal cryosections. A) and B) Transmitted light imaging performed on two separate sections. Letters indicate body muscles (b.m.), uterus (u) and intestine lumen (i). C) and D) Localization of the muscles using primary antibodies raised against myosin protein and stained with secondary, Alexa 594-labeled antibodies (red). E) Staining of UNC-29.1 showing expression in the body muscle as well as the uterus external muscular layer. F) Staining of UNC-29.2 revealing a similar localization to UNC-29.1. G) and H) Merge of green (UNC-29.1 or UNC-29.2) and red (myosin) signals. I–P) Confocal microscopy performed on male 10 μm transversal cryosections. I) and J) Transmitted light imaging performed on two separate sections. K) and L) Localization of the muscles with Alexa 594 secondary antibody (red). M) Staining of UNC-29.1 showing exclusive expression in the body muscles. N) Staining of UNC-29.2 revealing a similar localization. O) and P) Merge of UNC-29 copy (green) and myosin (red) signals. All slices are representative of the middle of the worm body and scale bars correspond to 100μm

Sections of adult male and female *H*. *contortus* worms revealed co-localization of both Hco-UNC-29.1 and Hco-UNC-29.2 with immunolabelled myosin heavy chain (Fig 8G, 8H, 8O and 8P). This immunoreactivity in myosin-associated tissues was observed in the body muscle cells and fluorescence was also detected in the muscles surrounding the uterus in females, indicating a potential role in the reproductive system (Fig 8E and 8F). In males, expression was also observed in body muscle, similar to females (Fig 8M and 8N). No specific signals were observed in any of the controls used in this experiment.

Co-expression of both UNC-29.1 and UNC-29.2 in the same *H*. *contortus* muscle tissue raised the possibility that they could interact *in vivo*. Although no functional L-AChR was produced in combination with UNC-29.2 alone, there remained the possibility that UNC-29.1 and UNC-29.2 may combine within a single receptor. Typically, such a situation can be identified by a change in the pharmacological properties of a receptor with addition of the second subunit [31–33]. To address this, we examined the possibility that UNC-29.2 may interfere with or modify the properties of the reconstituted L-AChR1.1. We prepared oocytes injected with cRNAs to express the *H*. *contortus* L-AChR1.1 and supplemented this with an increasing (0.2, 1, 5 times) concentration of cRNA for UNC-29.2 relative to UNC-29.1. The presence of unc-29.2 cRNA had no effect on the pharmacological response to either ACh or LEV nor in the observed current (S5 Fig). We can not rule out the possibility that UNC-29.2 combines into a receptor with UNC-29.1, but if it does, it has no detectable effect on the pharmacology or response of the receptor.

## Discussion

The free living nematode *C*. *elegans* was chosen as a model specifically amenable to the study of multicellular development and neuromuscular coordination [34]. One feature of *C*. *elegans* with particular relevance for the neuromusculature is the large number of genes encoding subunits of the pentameric ligand-gated ion-channels (pLGICs) that mediate fast synaptic neurotransmitter signalling [35, 36]. Expansion of the pLGIC family within the nematodes is characterized by gene duplication, associated with the development of new functionality as well as of subunit loss [10, 11, 32, 37–39]. The *C*. *elegans* model has proved particularly effective in the identification of specific anthelmintic drug targets in the parasitic nematodes, including the pLGICs mediating sensitivity to ivermectin, nicotine, levamisole and monepantel [3, 4, 40–42]. Although cognate parasite receptors exist, the precise details of the subunits contributing to the various receptors is different in almost every case examined [14, 15, 43–46]. This picture also includes a new class of parasite receptors specifically targeted by morantel, that is absent from *C*. *elegans* [47]. Understanding the biological context in which such receptor composition changes occur provides a means for understanding, and predicting, anthelmintic efficacy for a wide range of parasitic diseases. This would also provide a means for understanding the mechanisms behind the great diversification of neurotransmitter receptors that are a major feature of the nematode nervous system.

Three major classes of pLGIC are found within the same neuromuscular synapse of *C*. *elegans*, the stimulatory N-AChR and L-AChR, specifically activated by nicotine and levamisole respectively, and the inhibitory GABA gated chloride channel, UNC-49 [2]. These three display a range of diversification, from the homomeric N-AChR, the heteromeric UNC-49 where two principle subunit types are encoded by alternatively spliced transcripts [3, 31, 48] and the extreme L-AChR encoded by five separate genes. A previous study suggests that diversification of the L-AChR is recent, with duplication of the *unc-29* gene occurring since the divergence of the trichostrongylid parasitic nematodes from the common ancestor with *C*. *elegans* [16]. This suggests a means to dissect the conditions under which such diversification can occur and through characterization of new receptors, reveal the mechanisms used to bring about change. With this goal in mind, we set out to determine if the L-AChR meets the characteristics required for an effective model system. Such a model should be a specific example of a wider phenomenon, the duplications involved should be sufficiently recent that sequence substitutions responsible for functional change are identifiable with evidence that functional change has in fact occurred. We believe we have demonstrated that duplication of the *unc-29* gene within the *Trichostrongyloidea* meets these three criteria.

The phylogeny in Fig 1, together with the gene maps in Fig 2 include nine independent gene duplications events that occurred more recently than divergence of the *Strongylida* from the *Caenorhabditis* group. It is not yet possible to place a date on this event with any confidence, but Cutter estimates certainly more than 30 MYr based on analysis of neutral substitution rates [49]. The first of these duplications occurred prior to diversification within the *Strongylida* and the most recent occurring within *N*. *brasiliensis* after divergence from its neighbouring species. The four copies of *unc-29* within the *Trichostrongylidae* can therefore be considered as representative of the phenomenon in general.

Overall protein identity among copies of *unc-29* and within the clade V nematodes found up to 26% of amino acid positions were not conserved, with most substitutions occurring within the intracellular loop between TM3 and TM4 of the receptor subunit. The level of nucleotide substitution among copies of *unc-29* is well below the level required to reach saturation, suggesting that it will be possible to identify specific sequence changes that correlate with any change in function [50]. Relative substitution rate analysis identified significant increases in the rate at which amino acid replacement occurs for each, and only those branches deriving from a gene duplication event, consistent with the idea that a functional shift has occurred in each case. Individual amino acid sites that show evidence for positive selection (ω > 1) occurred within the intracellular TM3-TM4 loop or at positions where two subunits of the receptor could come into contact. This might suggest that functional changes coincided with modified subunit assembly and interaction. We would therefore have predicted that reconstituted receptors may show differences in their ability to co-assemble and in the maximal current observed in the presence of ACh.

The *Caenorhabditis* group *unc-29* and the duplicate copies found within the *Strongylida* share a common origin within the clade V nematodes. A first step in determining if there has been any functional divergence among *unc-29* copies is to determine if these are functionally equivalent. Decreased sensitivity to LEV in *C*. *elegans* carrying the definitive *unc-29* mutant (e1072) can be rescued by transgenic reintroduction of the *Cel-unc-29* gene [29]. We have shown here that the same can be achieved by reintroduction of any one of the four copies of *unc-29* from *H*. *contortus* (Fig 5). We also confirmed that this can be explained by co-assembly of the parasite derived protein with its *C*. *elegans* counterparts (Fig 6). This places an apparent upper limit on the degree of functional shift, since all four copies retain the ability to interact and co-assemble with the other subunits of the *C*. *elegans* L-AChR.

The exact composition of pentameric ligand-gated ion-channels *in vivo* is difficult to determine beyond question. The L-AChR of *C*. *elegans* is a special case where five different subunit genes are required to make up the receptor and each subunit is essential. Null mutants in any one of these subunit genes produces LEV resistance *in vivo* [51]. Even so, there remains the possibility that another class of L-AChR exists in *C*. *elegans* where LEV-8 may be replaced by the closely related ACR-8 [52]. Single channel conductance values for the native, and *Xenopus* reconstituted, *C*. *elegans* L-AChR are the same, confirming the native channel is equivalent to the reconstituted one [53, 54]. Reconstitution of parasite-derived L-AChRs has shown a remarkable variety of possible subunit compositions [13–15]. In the case of *O*. *dentatum*, one of the possible subunit combinations reconstituted in *Xenopus* oocytes does possess the same channel conductance properties as the native L-AChR *in vivo* [15]. It is not clear if the other combinations also represent channels that exist *in vivo* or not. We can not currently determine, with any certainty, the nature of the native *H*. *contortus* L-AChR *in vivo*. We have therefore focused our attention on the demonstration of any functional change in reconstitution of an L-AChR1 from *H*. *contortus* in *Xenopus* oocytes.

ACh affinity was very similar for the three *H*. *contortus* receptors reconstituted successfully (Fig 7 Table 2). This makes sense if functional change requires an ongoing, appropriate response to the native neurotransmitter. We were able to use this fact as a means to compare the different receptors to each other, standardizing to the ACh response. Each of the four *unc-29* paralogs showed distinct differences in terms of the maximal observed current induced by ACh, their affinity for, and response to, LEV and the profile of inhibition by Meca (Table 4). The reduced maximal current observed for *unc-29*.*4* is consistent with less efficient assembly of the receptor within the oocyte expression system or some inherent incompatibility within the complex, but we can not rule out the possibility that this could also be caused by alteration in the receptor's gating or conductance characteristics. The situation with *unc-29*.*2* was more extreme. Overall, we were able to confirm in each case that functional divergence between the copies had in fact occurred.

**Table 4:** Summary of functional differences between *H*. *contortus unc-29* copies.

	Hco-UNC-29.1	Hco-UNC-29.2	Hco-UNC-29.3	Hco-UNC-29.4
**Receptor**	L-AChR1.1	N/A	L-AChR1.3	L-AChR1.4
**Composition**	UNC-63	UNC-63	UNC-63	UNC-63
	UNC-38	UNC-38	UNC-38	UNC-38
	ACR-8	**ACR-8**	ACR-8	ACR-8
	**UNC-29.1**	**UNC-29.2**	**UNC-29.3**	**UNC-29.4**
**Max ACh Current**	~1.5 μA	0 μA	~1.5 μA	~500 nA
**Relative response**	LEV > ACh	N/A	LEV < ACh	LEV = ACh
**MECA**	Full Inhibition	N/A	Partial Inhibition	Full Inhibition
**Replace Cel-UNC-29 *in vivo***	YES	YES	YES	YES
**Replace Cel-UNC-29 in oocytes**	YES	YES	YES	YES
**Tissue localization**	Body muscle	Body muscle	N/A	N/A
	Uterus	Uterus		

An important open question concerns the role of the UNC-29.2 *in vivo*. Indeed, *unc-29*.*2* was the subunit that showed features of particular interest. Substitution rate analysis suggested that positive selective change occurred for amino acid sites distinct from the other copies. The complete lack of response for an *H*. *contortus* L-AChR1 containing UNC-29.2 was surprising, given the successful rescue of *unc-29* function in transgenic *C*. *elegans* and the successful reconstitution of a functional receptor with *C*. *elegans* subunits. Co-localization of UNC-29.1 and UNC-29.2 in the muscle tissue suggested that UNC-29.2 may be taking on a distinct role within the L-AChR1 although the fact that we found no evidence for an effect of UNC-29.2 on L-AChR1 function in terms of a change in pharmacology, nor a dominant negative effect on receptor expression argue against this [32, 33, 55, 56]. Alternatively, it is tempting to speculate that some mechanism has evolved to prevent an association between UNC-29.2 and the other L-AChR subunits in *H*. *contortus*. Such an incompatibility would have to be encoded in the sequence of the other *H*. *contortus* subunits, since replacing them with their *C*. *elegans* counterparts produces a functioning L-AChR containing UNC-29.2. In this scenario, UNC-29.2 would combine with other subunits entirely to produce a different class of receptor although to date we have no evidence to indicate what these may be.

This study provides a foundation for understanding the plasticity in subunit composition observed between pLGIC receptors in *C*. *elegans* and their counterparts in the parasitic nematodes, a central problem in understanding the mechanisms of anthelmintic activity for different human, animal and plant diseases [4, 13–15]. Sequence evidence supports the hypothesis that positive, directional selection is a significant force in generating neurotransmitter receptor subunit diversification and suggests this information may be used to investigate the mechanisms responsible for changing receptor composition and the fundamental process of receptor assembly and function.

## Methods

### Phylogeny and substitution rate analysis

Coding sequence orthologous to *Cel-unc-29* was identified from genome data available from the ParaSite WBPS2 release for clade III and clade V nematodes [6, 8]. These were *A*. *suum* (Asu), *T*. *callipaeda* (Tzc), *B*. *malayi* (Bma), *L*. *loa* (Llo), *D*. *immitis* (Dim), *O*. *volvulus* (Ovo), *L*. *sigmodontis* (Lsi), *A*. *viteae* (Avi) and *P*. *pacificus* (Ppa), *P*. *exspectatus* (Pex), *C*. *tropicalis* (Ctr), *C*. *japonica* (Cjp), *C*. *remanei* (Cre), *C*. *brigsgae* (Cbr), *C*. *elegans* (Cel), *H*. *contortus* (Hco), *T*. *colubriformis* (Tco), *T*. *circumcincta* (Tci), *A*. *costaricensis* (Acs), *D*. *viviparus* (Dvi), *O*. *dentatum* (Ode), *A*. *caninum* (Aca), *A*. *ceylanicum* (Ace) and *N*. *brasiliensis* (Nbr). Sequences were aligned as codons using the MAFFT plugin (v1.3.3) of Geneious (v7.1.2, Biomatters Ltd) [57]. Regions corresponding to the signal peptide, the intracellular loop between TM3 and 4, the C-terminal tail beyond TM4 and any position containing a gap, were removed. A maximum likelihood phylogeny was determined using PhyML (v20120412) with a discrete gamma model and 20 site rate categories, the reversible nucleotide substitution model 012012 (see below), branch length, topology and rate parameter optimization. The optimal tree was identified as the best of both NNI and SPR searches based on an initial neighbour-joining tree and nine random starting trees. The analysis was repeated 12 times with different starting conditions to avoid local maxima in the likelihood surface. Branch support was evaluated from SH statistics. [58].

Relative substitutions rates, branch lengths and transition/transversion ratios were estimated using codeml (PAML v4.8) based on the unrooted tree shown in Fig 3 and codon alignment (Fig 4) [21]. Models were evaluated with a single substitution rate across all branches and residues (Model 0), Model 3, with either 2 or 3 different rate categories (M3k2 and M3k3) and various branch-site models D with two fixed site rate classes shared across all branches of the tree and a single site rate class that could vary over marked branches [59]. Significant improvement of various nested models was determined from twice the difference in log likelihood (2δ) assuming a chi-square distribution and degrees of freedom of the different is the number of free parameters [21].

The remaining substitution rate analysis was carried out using HyPhy v2.2.6, with the same alignment and phylogenetic tree as above [24]. The best of the 203 reversible nucleotide substitution models to fit the data (model 012012) was determined from the NucModelCompare.bf script and used for the PhyML and subsequent HyPhy analysis. Substitution rates for each codon site were estimated using the two site FELS analysis implemented in HyPhy v2.2.6 and sites under positive selection for marked branches were taken to be significant with a probability less than 0.1.

### Ethics statement

Frogs (*Xenopus laevis*) and sheep were maintained under Animal Use Protocols #AUP5284 and #AUP3845, respectively, approved by the Macdonald Campus Facility Animal Care Committee of McGill University. These protocols adhered to the standards set out by the Canadian Council on Animal Care in Science. *Xenopus laevis* oocytes were surgically removed from adult female frogs by certified personnel following guidelines laid out in the AUP. Sheep were infected with approximately 5,000 infective L3 larvae by oral gavage and adult parasites collected at necropsy.

### Nematodes

Adult, L2 and L3 *H*. *contortus* from the laboratory isolate PF23, an anthelmintic sensitive isolate, were used for immunofluorescence studies [60]. Adult male and female nematodes were collected 30 days after infection, from the abomasal mucosa of sheep at necropsy. Parasite eggs were cultured into L2 or L3 larvae from faeces stored at 18°C. *C*. *elegans* wild-type Bristol N2 and CB1072:e1072 were obtained from the *Caenorhabditis* Genetics Center, funded by the National Institute of Health National Center for Research Resources.

### Accession numbers

cDNA clones used in this study correspond to sequences, *Cel-lev-1* (NP_502534), *Cel-lev-8* (NP_509932), *Cel-unc-29* (NP_492399), *Cel-unc-38* (NP_491472) and *Cel-unc-63* (NP_491533); *Hco-unc-29*.*1* (GU060980), *Hco-unc-29*.*2* (GU060981), *Hco-unc-29*.*3* (GU060982), *Hco-unc-29*.*4* (GU060983), *Hco-unc-38* (GU060984), *Hco-unc-63a* (GU060985), *Hco-acr-8* (EU006785), *Hco-unc-50* (HQ116822), *Hco-unc-74* (HQ116821), *Hco-ric-3*.*1* (HQ116823).

### Drugs

Acetylcholine chloride (ACh), BAPTA-AM, bephenium hydroxynaphtoate, 1,1-dimethyl-4-phenylpiperazinium iodide (DMPP), mecamylamine hydrochloride, morantel citrate, (-)-tetramisole hydrochloride (levamisole), (-)-nicotine hydrogen tartrate, pyrantel citrate, (+)-tubocurarine chloride hydrate (dTC) were purchased from Sigma-Aldrich. Dihydro-berythroidine hydrobromide (DHβE) was obtained from Tocris Bioscience.

### *Xenopus* oocyte electrophysiology

The *H*. *contortus* cDNAs encoding AChR subunits and ancillary proteins were available as expression clones in the pTB207 vector and *C*. *elegans* expression clones were a gift from T. Boulin. [4, 14]. cRNA synthesis of each construct required *Nhe*I-linearized templates and was performed with the mMessage mMachine T7 Kit (Ambion) according to the manufacturer’s instructions. cRNAs were precipitated with lithium chloride and redissolved in RNAse-free water and stored at -80°C. cRNA concentration was determined using a Nanodrop spectrophotometer (Thermo Scientific) and cRNA purity and integrity was assessed by electrophoresis through a 1% gel stained with GreenGlo nucleic acid dye (Denville Scientific Inc.).

Oocytes were collected from mature *Xenopus* females according to standard procedures [61] and incubated at 19°C in a ND96 solution (NaCl 96 mM, KCl 2 mM, CaCl_2_ 1.8 mM, MgCl_2_ 1 mM, and HEPES 5 mM, pH 7.3 supplemented with sodium pyruvate 2.5 mM) [43]. Defolliculated oocytes were injected into the animal pole with approximately 36 nL of a cRNA mix containing 50 ng/μL of each cRNA in RNAse-free water using the Nanoject system (Drummond Scientific, Broomall, PA). Negative controls consisted of oocytes injected with RNAse-free water. Oocytes were then stored at 19°C in ND96 for 5 days to allow receptor expression. In order to prevent activation of endogenous calcium-activated chloride channels, the oocytes were incubated in ND96 supplemented with 100 μM of the calcium chelator BAPTA-AM, 4h prior to recording [4].

Two-electrode voltage-clamp experiments were carried out using a perfusion system connected to a flat RC-1Z chamber (Harvard Apparatus). Glass electrodes were filled with 3M KCl and checked for resistance between 1 and 3 MΩ. The oocyte bath was connected to the ground through a 3 M KCl agar bridge. Agonist and antagonists were dissolved or diluted in recording solution (100 mM NaCl, 2.5 mM KCl, 1 mM CaCl_2_, 5 mM HEPES, pH 7.3). All measurements were performed using a Geneclamp 500B amplifier and Digidata 1322M (Axon Instruments), oocytes were voltage-clamped at -60 mV. Drug solutions were applied to oocytes by gravity flow using perfusion syringes directly connected to the chamber. Data were collected and analyzed using Clampex 9.2 and Clampfit 9.2 (Axon Instruments, Sunnyvale, CA, USA). Traces were filtered at 30 Hz and analyzed using GraphPad Prism 5.0 software (GraphPad Software, San Diego, CA, USA). Final results are presented as means ± SE.

In all recordings, the peak currents in response to applied drugs were measured and normalized to the response elicited by 100 μM ACh and expressed as the mean ± S.E. Dose-response relationships were analyzed by fitting log dose-response data points with the Hill equation using GraphPad Prism 5.0 software (GraphPad Software, San Diego, CA, USA) as previously described [4].

### *C*. *elegans* microinjection

The *Cel-unc-29*, *unc-29*.*1*, *unc-29*.*2*, *unc-29*.*3*, *unc-29*.*4* cDNA sequences were amplified by PCR using primers containing restriction site inserted with restriction enzymes in the plasmid backbone (Addgene L2534) containing the myo-3 promoter. Strains used in these study were the wild-type Bristol isolate N2 and the LEV resistant isolate CB1072: *unc-29 (e1072)*. Worms were grown on *Escherichia coli* OP50 seeded NGM (Nematode Growth Medium) plates at 20°C and maintained using standard culture methods [34]. Hermaphrodite young adult *C*. *elegans* were transformed by microinjection of plasmids into the gonads [62]. Transformation was accomplished by co-injecting the plasmid vector (Addgene L2534: 30 ng/μl) containing each *Hco-unc-29* cDNA sequences driven by the *myo-3* promoter and *the ttx-3*::GFP-expressing plasmid [63] at identical concentration. Successful transformation was determined by the observation of fluorescence of the selection marker in the two targeted neurones of the head region. Only worms carrying the selection marker were used for the behavioural and pharmacological analysis.

### *C*. *elegans* paralysis assay

Three day old adult fluorescent worms were transferred onto unseeded NGM plates, equilibrated at 20°C, containing 200 μM LEV. Drug induced paralysis was determined by visual inspection every 15 min. Worms were defined as paralyzed if no movement was observed within five seconds after prodding the tail with a hair tip [52]. Statistics. Experimental data are shown as mean ± S.D. Statistical comparisons between the *unc-29 (e1072)* mutant strain and the transformed lines were done using two-way ANOVA with Bonferroni’s multiple comparison post test. A level of p < 0.05 was considered significant.

### Antibody production

Peptide-derived polyclonal antibodies were generated in rabbits against subunits UNC-29.1 and UNC-29.2, in rats (21st Century Biochemicals—Marlborough, MA). Animals were immunized with a mixture of two specific peptides per subunit corresponding to parts of the intracellular loop located between the third and the fourth TM domains. For UNC-29.1, the synthetic peptides were "EKTASPKTLNCAMELTTRDPQL" and "PIPEPAPPEITQLP" The UNC-29.2 peptides corresponded to "VSIKVLNCGDDIPKDP" and "MTKKKRGATVAKLP" sequences. Purified antibodies were tested for specificity and titrated against both peptides by ELISA.

### Immunohistochemistry on *Xenopus* oocytes

Oocytes were collected, treated and injected as previously described for receptor reconstitution. Approximately 12 oocytes were injected per subunit mix. After 5 days, TEVC was performed to verify AChR expression with exposure to 100 μM ACh. Oocytes were then incubated overnight in 1X PBS with 4% paraformaldehyde (PFA) at 4°C. After being washed 3 times, 5 min each in 1X PBS, oocytes were then blocked overnight in an antibody diluent (AbD) containing 1X PBS, 0.2% gelatin from cold water fish skin, 2.5% Triton X-100 and 0.1% sodium azide. Primary antibodies were diluted at 1:100 in AbD and applied on oocytes overnight at 4°C. The eggs were washed 3 to 4 times in AbD and incubated with the corresponding 1:500 secondary antibodies conjugated to Alexa Fluor 488 (Invitrogen, USA) overnight at 4°C. Final washes were performed 2 times with AbD and 2 times with 1X PBS/0.1% (v/v) Triton X-100. The membrane of the oocytes was observed using a Zeiss LSM710 confocal microscope (Carl Zeiss Inc., Canada) equipped with the Zeiss Zen 2010 software. Negative controls were either water-injected oocytes or AChR-expressing oocytes but omitting the primary antibodies.

### Immunohistochemistry on adult *H*. *contortus* sections

Male and female PF23 *H*. *contortus* were extracted and fixed overnight in PBS 1X supplemented with 4% PFA at 4°C. The worms were washed 3 times in PBS 1X and incubated overnight at 4°C in PBS 1X with 30% sucrose under gentle rocking. The nematodes were rapidly frozen in dry ice after embedding in an optimal cutting temperature compound (OCT) (Thermo Fisher Scientific, USA). Cryosections were performed transversally using a Thermo Shandon cryotome (Thermo Fisher Scientific, USA). 10 μm slices were placed on microscope slides and blocked overnight in AbD. Worms sections were then independently incubated overnight at 4°C with a 1/100 dilution of each specific anti-Hco-UNC-29 antibody. After washing a minimum of five times with AbD at 4°C, the sections were incubated overnight with the corresponding 1:500 secondary antibodies conjugated to Alexa Fluor 488 (Invitrogen, USA). Final washes were performed two times in AbD and 2 times in 1X PBS/0.1% (v/v). Slides were then mounted using a Mounting medium (Sigma) and examined with the same confocal microscope.

The omission of the primary antibodies as well as the use of peptide adsorbed primary antibodies were used as controls. In the latter case, primary antibody were pre-adsorbed with 0.25 mg/ml of the corresponding peptide antigen.

## Supporting Information

S1 Fig Reconstitution and pharmacological characterization of L-AChR1.2 in *Xenopus* oocytes. TEVC experiments were performed in *Xenopus* oocytes injected with *Hco-unc-63*, *Hco-unc-38*, *Hco-acr-8*, *Hco-unc-29*.*2* and *Hco-ric-3*.*1*, *Hco-unc-74*, *Hco-unc-50* cRNAs. Representative recording trace from a single oocyte perfused with 100μM of the following cholinergic agonists: acetylcholine (ACh), Dimethylpiperazinium (DMPP), Pyrantel (PYR), Nicotine (NIC), Bephenium (BEPH) and Levamisole (LEV).
(TIF)

S2 Fig ACh response of *C*. *elegans* / *H*. *contortus* AChR subunit admixtures in *Xenopus* oocytes. TEVC experiments were performed on oocytes injected with *Cel-unc-63*, *Cel-unc-38*, *Cel-lev-8*, *Cel-lev-1* cRNAs and co-expressed with *H*. *contortus* accessory proteins, *ric-3*, *unc-50* and *unc-74*. The addition of cRNAs encoding *Cel-unc-29*, *unc-29*.*1*, *unc-29*.*2*, *unc-29*.*3* and *unc-29*.*4* were tested independently. Current (nA) evoked with 100 μM ACh recorded from the different subunit combinations is indicated above each column. Error bars indicate SD.
(TIF)

S3 Fig Pharmacological profiles of L-AChR1.1, L-AChR1.3 and L-ACR1.4. All drugs were applied at a concentration of 100μM. All values are normalized to the current evoked by perfusion of 100μM ACh. Statistics. Experimental data are shown as mean ± SE. Statistical comparisons were done using one-way ANOVA with Bonferroni’s multiple comparison post test. Asterisks, ***, indicate a significant difference between receptors (p<0.001).
(TIF)

S4 Fig Addition of unc-29.2 to the L-AChR1.1 in *Xenopus* oocytes. TEVC experiments were performed on *Xenopus* oocytes injected with *Hco-unc-63*, *Hco-unc-38*, *Hco-acr-8*, *Hco-unc-29*.*1* and *Hco-ric-3*.*1*, *Hco-unc-74*, *Hco-unc-50* cRNAs. *Hco-unc-29*.*2* cRNA was co-injected at 0.2, 1 or 5 times the concentration of *unc-29*.*1*. Dose-response curves are shown for each ratio for ACh (red) and LEV (blue). All responses are normalized to 100μM ACh, which corresponds to the saturating dose. The ACh and LEV 50% effective concentration (EC50) values as well as Hill coefficients are indicated in S1 Table. Errors bars represent SD.
(TIF)

S5 Fig Lack of cross-reactivity between antibodies against UNC-29.1 and UNC-29.2. *Xenopus* oocytes were injected with the L-AChR1.1 and L-AChR1.2 corresponding cRNA mixtures. After 5 days, oocytes were checked for expression using the TEVC technique (when applicable), fixed and incubated with affinity-purified antibodies raised against Hco-UNC-29.1 and Hco-UNC-29.2 specific peptides. Localization of subunits was performed using Alexa 488-labeled secondary antibodies (green). Controls were un-injected oocytes incubated in anti-UNC-29.1 antibodies and L-AChR1-expressing oocytes incubated with the secondary antibodies only. Confocal microscopy was performed on the whole oocytes. All slides were observed under 20x magnification. Scale bars correspond to 100 μm.
(TIF)

S1 Table ACh and LEV response profiles for increasing addition of UNC-29.2 to the L-AChR1.1 from S3 Fig. (DOCX)

S1 File **Zip archive containing: *unc-29 sequences*.*gb*:** Nucleotide sequences of *unc-29* used to prepare Fig 1. The position of the four transmembrane regions of each sequence along with the region trimmed to produce the alignment used for the substitution rate analysis are annotated. ***paml unc-29 phylogeny*.*newick*:** Newick format file used for the substitution rate analysis. ***paml unc-29 alignment*.*phy*:** Phylip format file of the sequence alignment used for the substitution rate analysis.
(ZIP)
