# Supplementary figures and images for: Recent Duplication and Functional Divergence in Parasitic Nematode Levamisole-Sensitive Acetylcholine Receptors
Source: PLoS Negl Trop Dis. 2016 Jul 14;10(7):e0004826. doi: 10.1371/journal.pntd.0004826 (PMC4945070; doi:10.1371/journal.pntd.0004826)

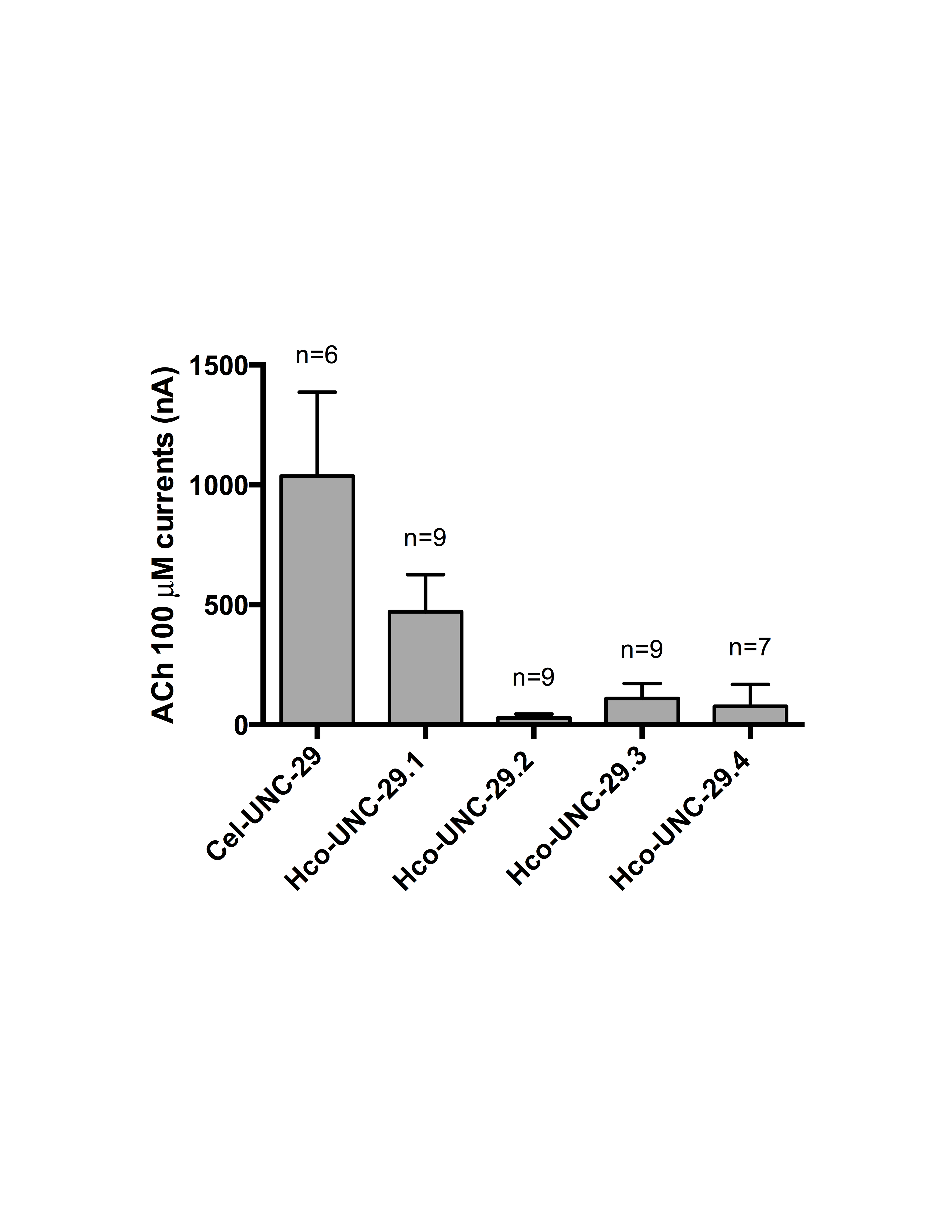

Supplement: S1 Fig — TEVC experiments were performed in Xenopus oocytes injected with Hco-unc-63, Hco-unc-38, Hco-acr-8, Hco-unc-29.2 and Hco-ric-3.1, Hco-unc-74, Hco-unc-50 cRNAs. Representative recording trace from a single oocyte perfused with 100μM of the following cholinergic agonists: acetylcholine (ACh), Dimethylpiperazinium (DMPP), Pyrantel (PYR), Nicotine (NIC), Bephenium (BEPH) and Levamisole (LEV). (TIF) [file pntd.0004826.s001.tif]

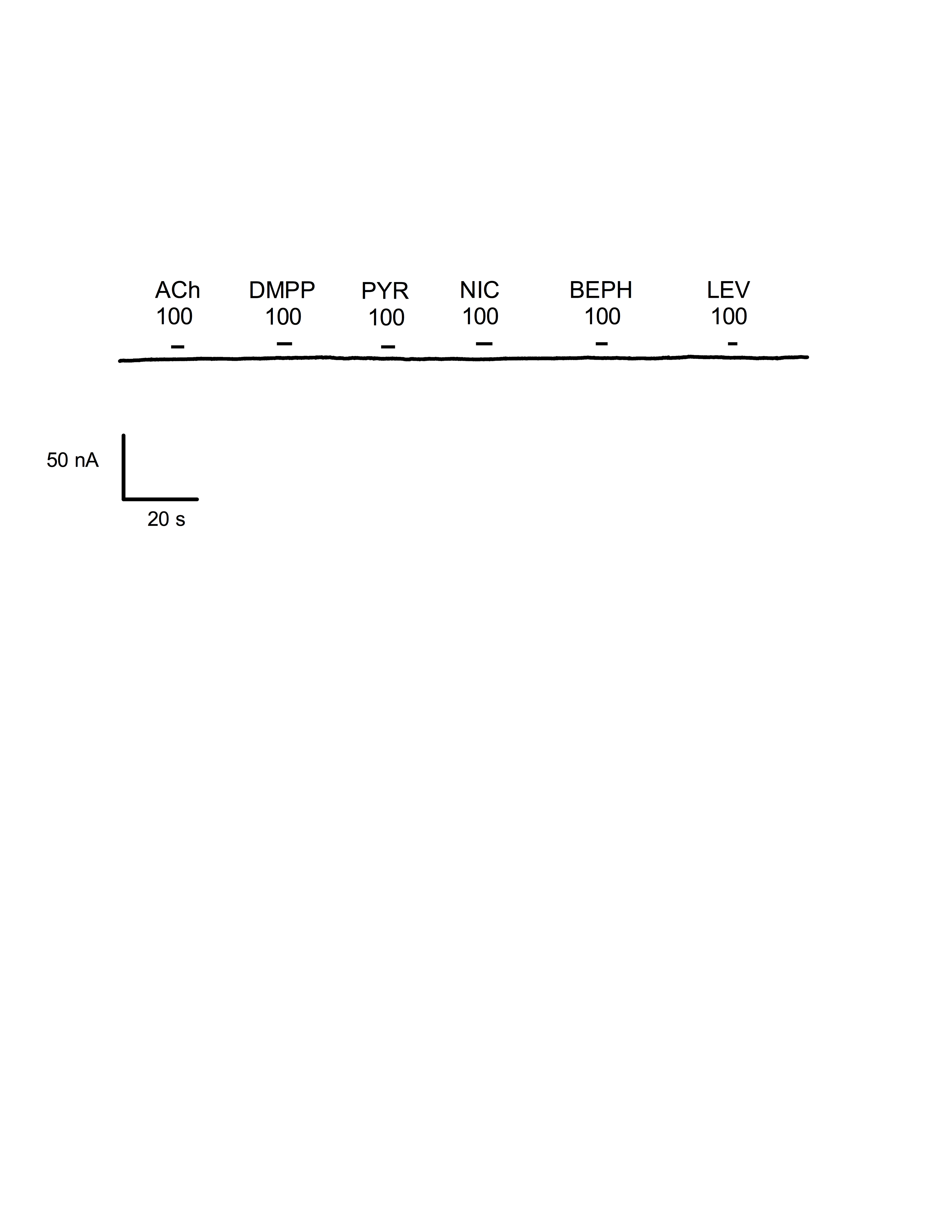

Supplement: S2 Fig — TEVC experiments were performed on oocytes injected with Cel-unc-63, Cel-unc-38, Cel-lev-8, Cel-lev-1 cRNAs and co-expressed with H. contortus accessory proteins, ric-3, unc-50 and unc-74. The addition of cRNAs encoding Cel-unc-29, unc-29.1, unc-29.2, unc-29.3 and unc-29.4 were tested independently. Current (nA) evoked with 100 μM ACh recorded from the different subunit combinations is indicated above each column. Error bars indicate SD. (TIF) [file pntd.0004826.s002.tif]

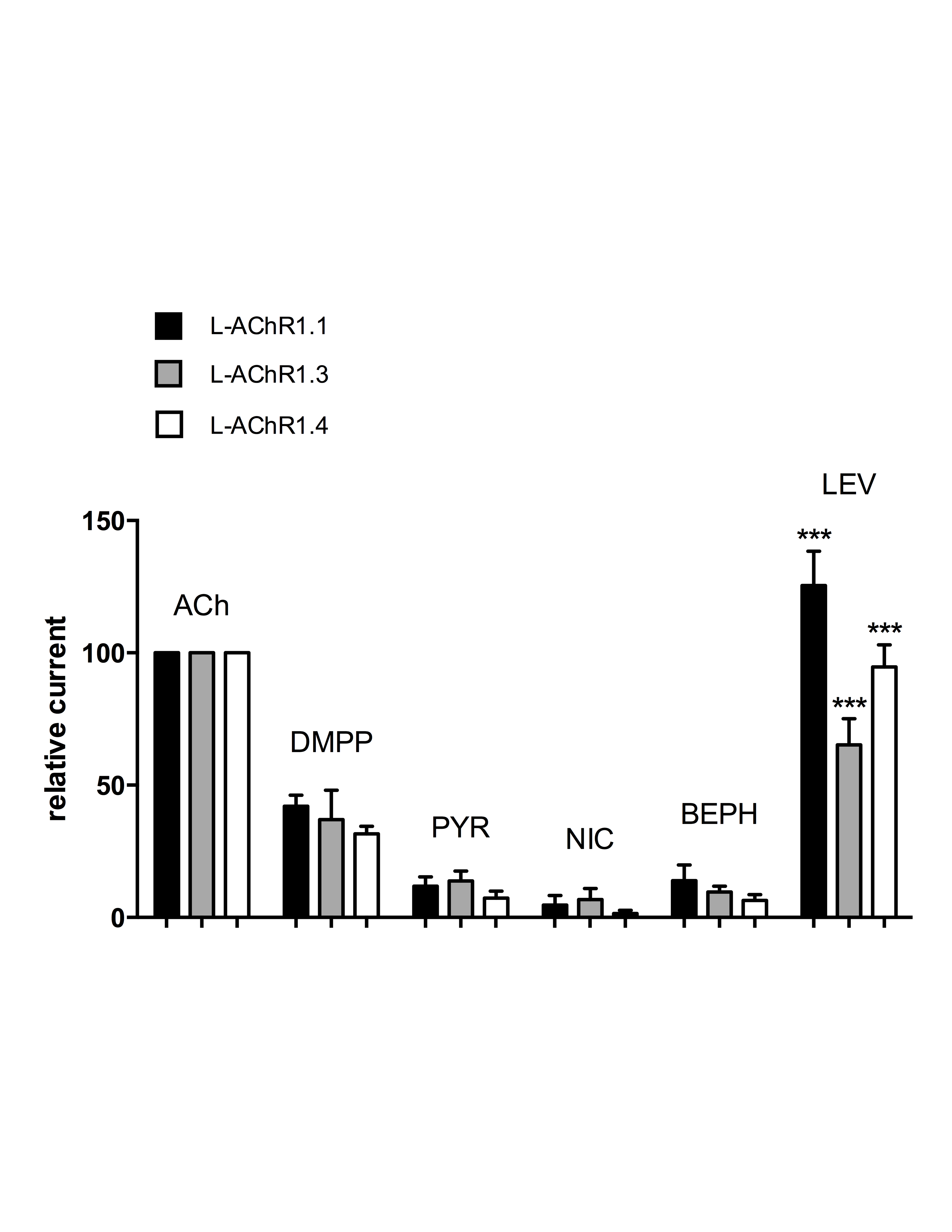

Supplement: S3 Fig — All drugs were applied at a concentration of 100μM. All values are normalized to the current evoked by perfusion of 100μM ACh. Statistics. Experimental data are shown as mean ± SE. Statistical comparisons were done using one-way ANOVA with Bonferroni’s multiple comparison post test. Asterisks, ***, indicate a significant difference between receptors (p<0.001). (TIF) [file pntd.0004826.s003.tif]

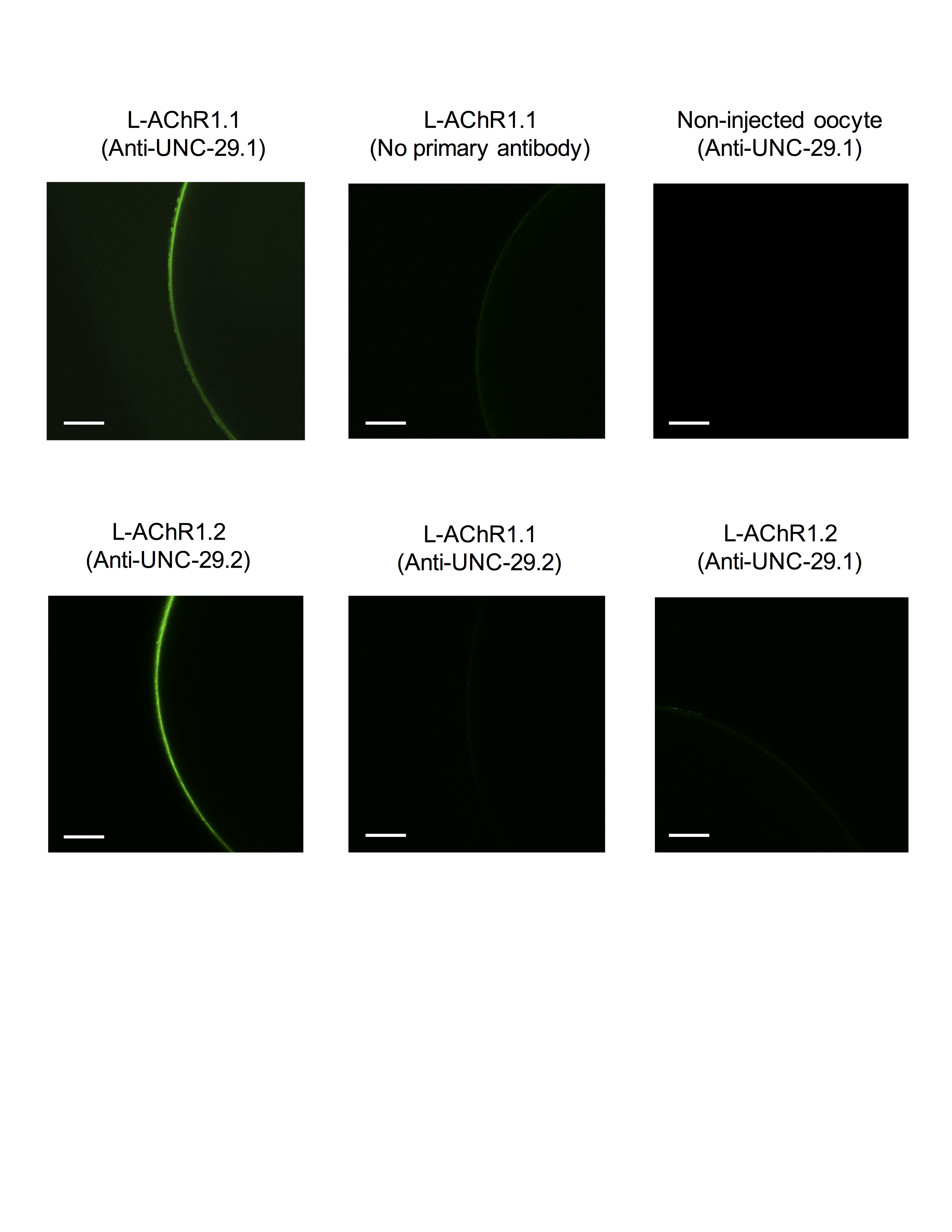

Supplement: S4 Fig — TEVC experiments were performed on Xenopus oocytes injected with Hco-unc-63, Hco-unc-38, Hco-acr-8, Hco-unc-29.1 and Hco-ric-3.1, Hco-unc-74, Hco-unc-50 cRNAs. Hco-unc-29.2 cRNA was co-injected at 0.2, 1 or 5 times the concentration of unc-29.1. Dose-response curves are shown for each ratio for ACh (red) and LEV (blue). All responses are normalized to 100μM ACh, which corresponds to the saturating dose. The ACh and LEV 50% effective concentration (EC50) values as well as Hill coefficients are indicated in S1 Table. Errors bars represent SD. (TIF) [file pntd.0004826.s004.tif]

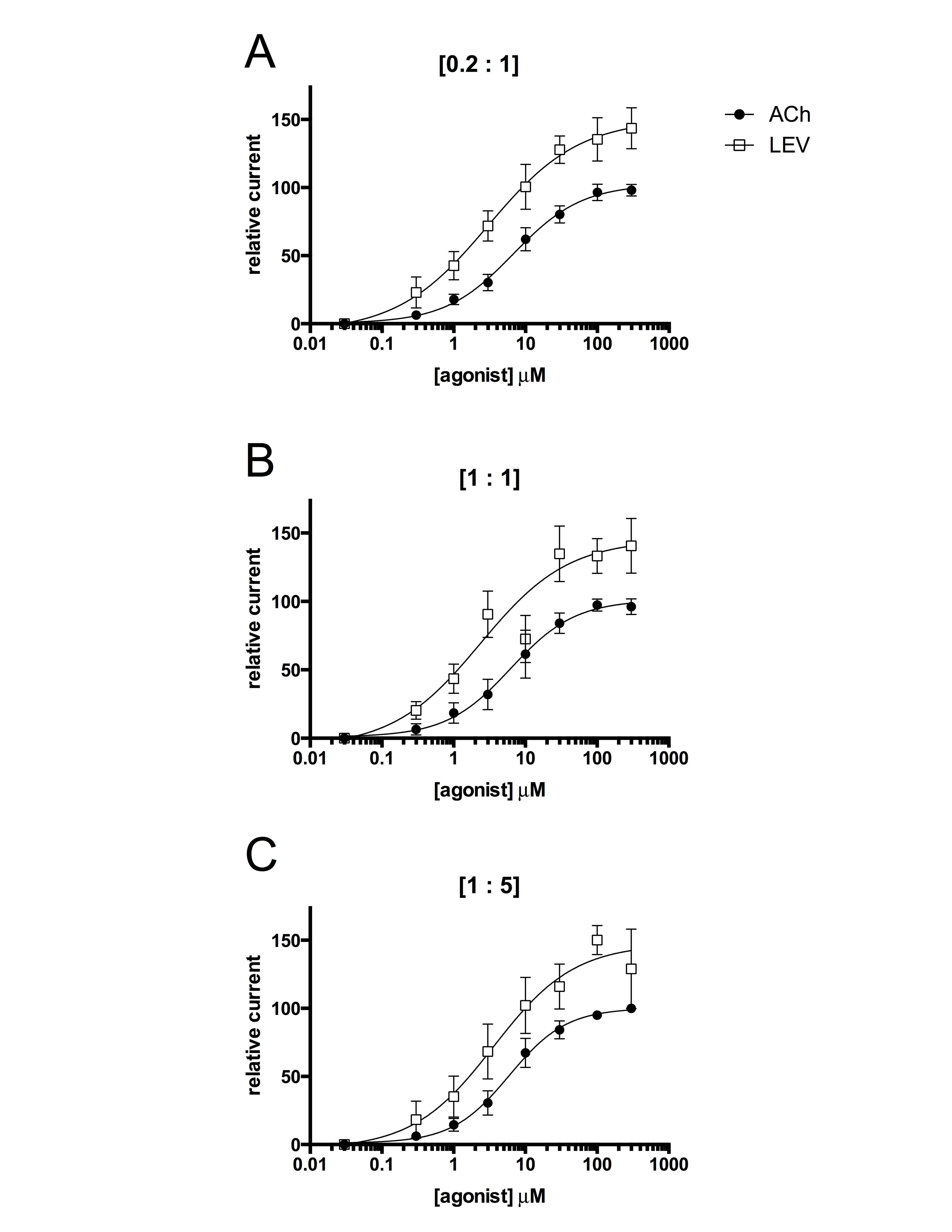

Supplement: S5 Fig — Xenopus oocytes were injected with the L-AChR1.1 and L-AChR1.2 corresponding cRNA mixtures. After 5 days, oocytes were checked for expression using the TEVC technique (when applicable), fixed and incubated with affinity-purified antibodies raised against Hco-UNC-29.1 and Hco-UNC-29.2 specific peptides. Localization of subunits was performed using Alexa 488-labeled secondary antibodies (green). Controls were un-injected oocytes incubated in anti-UNC-29.1 antibodies and L-AChR1-expressing oocytes incubated with the secondary antibodies only. Confocal microscopy was performed on the whole oocytes. All slides were observed under 20x magnification. Scale bars correspond to 100 μm. (TIF) [file pntd.0004826.s005.tif]
