# Supplementary material for: Recent Duplication and Functional Divergence in Parasitic Nematode Levamisole-Sensitive Acetylcholine Receptors
Source: PLoS Negl Trop Dis. 2016 Jul 14;10(7):e0004826. doi: 10.1371/journal.pntd.0004826 (PMC4945070; doi:10.1371/journal.pntd.0004826)
Supplement: S1 Table — (DOCX) [file pntd.0004826.s006.docx]

**Table S1 ACh and LEV response profiles for increasing addition of UNC-29.2 to the L-AChR1.1 from Fig S3.**

|  |  | **ACh** |  |  | **LEV** |  |
| --- | --- | --- | --- | --- | --- | --- |
| **29.1 : 29.2** | **EC_50_ (µM)** | **Hill** | **n** | **EC_50_ (µM)** | **Hill** | **n** |
| 1 : 0.2 | 6.82 ± 0.69 | 0.91 ± 0.08 | 6 | 3.05 ± 0.44 | 0.69 ± 0.09 | 6 |
| 1 : 1 | 6.33 ± 0.92 | 0.96 ± 0.13 | 7 | 2.41 ± 0.08 | 0.71 ± 0.13 | 7 |
| 1 : 5 | 5.96 ± 0.57 | 1.09 ± 0.10 | 6 | 3.70 ± 0.35 | 0.77 ± 0.13 | 6 |
